# Distinct promoter activation mechanisms modulate noise-driven HIV gene expression

**DOI:** 10.1038/srep17661

**Published:** 2015-12-15

**Authors:** Arvind K. Chavali, Victor C. Wong, Kathryn Miller-Jensen

**Affiliations:** 1 Department of Biomedical Engineering, Yale University 55 Prospect Street, New Haven, CT 06511; 2 Department of Molecular, Cellular, and Developmental Biology, Yale University 55 Prospect Street, New Haven, CT 06511

## Abstract

Latent human immunodeficiency virus (HIV) infections occur when the virus occupies a transcriptionally silent but reversible state, presenting a major obstacle to cure. There is experimental evidence that random fluctuations in gene expression, when coupled to the strong positive feedback encoded by the HIV genetic circuit, act as a ‘molecular switch’ controlling cell fate, i.e., viral replication versus latency. Here, we implemented a stochastic computational modeling approach to explore how different promoter activation mechanisms in the presence of positive feedback would affect noise-driven activation from latency. We modeled the HIV promoter as existing in one, two, or three states that are representative of increasingly complex mechanisms of promoter repression underlying latency. We demonstrate that two-state and three-state models are associated with greater variability in noisy activation behaviors, and we find that Fano factor (defined as variance over mean) proves to be a useful noise metric to compare variability across model structures and parameter values. Finally, we show how three-state promoter models can be used to qualitatively describe complex reactivation phenotypes in response to therapeutic perturbations that we observe experimentally. Ultimately, our analysis suggests that multi-state models more accurately reflect observed heterogeneous reactivation and may be better suited to evaluate how noise affects viral clearance.

Noise in gene expression can lead to detectable phenotypic heterogeneity between cells in an otherwise genetically identical population^1^. The origins of molecular noise are biochemical in nature and can result from intrinsic or extrinsic sources, including fluctuations in promoter transitions between inactive and active states, random timing of transcription or translation reactions, variations in cellular microenvironment, differences in cell size, asymmetric partitioning of cellular components following division, as well as fluctuations in abundances and availability of transcription factors, polymerases, and ribosomes^2^,^3^,^4^,^5^,^6^. In eukaryotic systems, the local chromatin environment at the promoter is thought to contribute significantly to gene expression noise^3^. A gene is able to transcribe relatively freely when the chromatin environment surrounding the promoter is in an open, acetylated configuration and nucleosomes are not occluding the transcription start site. However, transcription is repressed when the chromatin is in a condensed state^4^. Although direct evidence of chromatin remodeling as the primary cause of stochastic gene expression has proven elusive, several studies have indirectly linked chromatin-related events to stochastic gene expression^7^,^8^,^9^. Furthermore, recent electron microscopy observations in budding yeast have shown nucleosome configurations to be intrinsically stochastic^10^.

Here, we focused on a clinically relevant problem–latency in human immunodeficiency virus-1 (HIV) infection–wherein noise at the promoter is regulated by epigenetic features at the integration site^8^ and may have significant implications for disease outcomes^11^. For patients infected with HIV, latent reservoirs of infected resting memory CD4 ^+^ T cells escape detection by the immune system and are unaffected by highly active anti-retroviral therapy (HAART)–thus remaining one of the biggest obstacles to permanent cure^12^. Clinical research efforts have focused on an ‘activate-and-kill’ strategy to therapeutically reactivate the latent pool with small molecule drugs or cytokines that would result in death of the reservoir by host immune responses or by viral cytopathic effects^13^. However, recent experimental evidence from patient samples suggests that reactivation from a latent state may be a probabilistic phenomenon with latent proviruses remaining inactive despite maximal T cell stimulation^14^. Biological noise in viral transcription from the HIV promoter could be the cause of such probabilistic reactivation^15^, and therefore, the ultimate success of any strategy directed towards purging the latent reservoir may depend on countering the effects of noise generated by the HIV genetic circuit.

The HIV promoter has been put forward as a model for noisy gene expression that is influenced by its local epigenetic environment^3^. This promoter consists of a 5′ long-terminal repeat (LTR) region that contains a positioned nucleosome at the transcriptional start site, as well as binding sites for key regulators such as NF-κB and Sp1^12^,^16^. Experimental studies have revealed a critical role of nucleosome organization at the LTR and chromatin density at the site of integration in regulating noise^8^. However, for noise in gene expression to have phenotypic consequences, there must be a means of stabilizing differences in the cell population that arise stochastically^1^. Positive feedback provides a mechanism to amplify and stabilize stochastic fluctuations to produce bimodal and in some cases bistable populations. The HIV genome encodes for such a positive transcriptional regulator called *Tat*, or Trans-activator of transcription. Stochastic fluctuations in Tat expression play an essential role in the replication-versus-latency decision of proviruses because Tat is capable of powering a strong positive feedback loop that auto-stimulates its own expression 50- to 100-fold over basal levels^17^,^18^. These fluctuations drive phenotypic bifurcation, in which cells with low Tat and high Tat expression co-exist within clonal populations^19^,^20^. High Tat expression results in ‘productive’ viral replication whereas low Tat expression maintains ‘unproductive’ latent infections. Due to probabilistic initiation of transcription, there can be long delays prior to cells transitioning from an unproductive to a productive state^19^, which likely contribute to heterogeneous reactivation from latency^21^. Consequently, stochastic fluctuations in the underlying regulatory mechanisms controlling HIV gene expression can drive cells to distinct phenotypic states that may be related to viral latency.

If noise-driven gene expression underlies viral latency, then computational models that describe how regulatory mechanisms at the promoter affect heterogeneous viral activation could be used to assess treatment strategies focused on reducing or eliminating the latent reservoir. Here, we present a computational analysis of stochastic HIV gene expression coupled with Tat positive feedback for different promoter activation mechanisms. Through systematic evaluation of one-, two-, and three-state promoter models, we investigate how noise in protein production generated by these different promoter configurations–when coupled to the HIV genetic circuit–modulate heterogeneous reactivation from latency. We demonstrate that the greater parameter space afforded by mathematical models of transcription containing multiple promoter states can reproduce a range of experimentally observed behaviors following virus reactivation that are indicative of the numerous biological mechanisms that maintain latent infections.

## Results and Discussion

### Interaction between basal transcription and strength of positive feedback drives heterogeneous activation in a one-state promoter model

**Figure 1:**
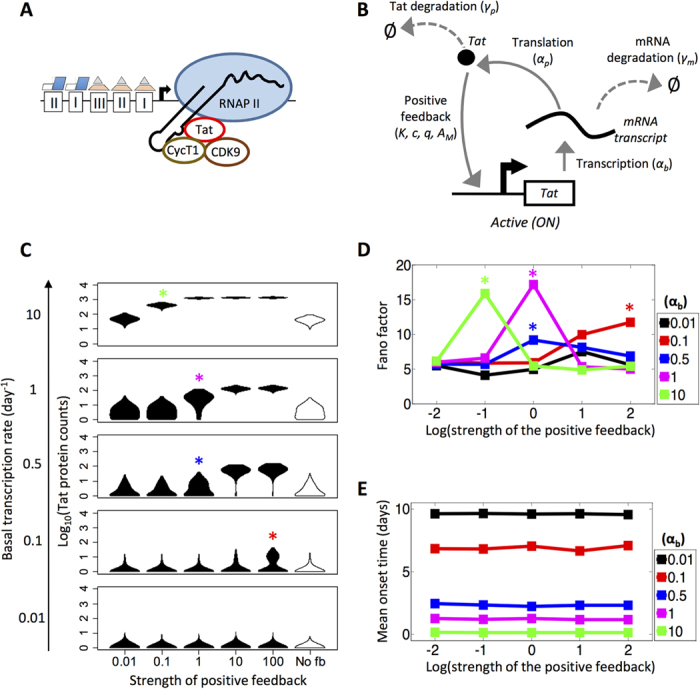
Varying basal transcription rate and strength of positive feedback affects protein heterogeneity in a one-state promoter model. **(A)** Conceptual schematic based on biological understanding of an ‘ideal’ promoter configuration for HIV reactivation as a fully active provirus with all transcriptional machinery available for sustained viral mRNA synthesis. **(B)** Schematic depiction of a one-state computational model with positive feedback. Reactions involving transcription, translation, degradation, and positive feedback are depicted. **(C)** Violin plots of the steady-state endpoint protein distributions for a range of basal transcription rates and positive feedback strengths. Violin plots capturing endpoint distributions for a system without feedback are shown on the far right (white shading; ‘No fb’). The stochastic model for every parameter set was run 1000 times for a period of 10 days. **(D)** Fano factors for the one-state model with positive feedback are shown for different basal transcription rates of 0.01 (black), 0.1 (red), 0.5 (blue), 1 (magenta), and 10 (green) across varying strengths of positive feedback. The x-axis is presented in log scale. **(E)** Mean onset times for the one-state model with positive feedback are shown for different basal transcription rates of 0.01 (black), 0.1 (red), 0.5 (blue), 1 (magenta), and 10 (green) across varying strengths of positive feedback. If a cell did not activate during the simulation, onset time was set to 10 days.

Biologically, we can imagine an ‘ideal’ promoter configuration for HIV reactivation as a fully active provirus with all transcriptional machinery available in order to induce sustained viral mRNA synthesis. Such a configuration is characterized by binding of NF-κB and Sp1 to their respective sites on the LTR, acetylated histones at the promoter, and a displaced nucleosome-1 (Nuc-1), such that the transcription start site is accessible for continuous binding and initiation of transcription by RNA polymerase II (RNAPII) (Fig. 1A)^16^,^22^. Mathematically, the simplest network that permits us to simulate this configuration is a model with a single promoter state (termed ‘one-state model’) incorporating equations for transcription, translation, degradation, and Tat-mediated positive feedback (Fig. 1B).

To explore the parameter space of this one-state model, we first asked how the dynamics of transcription would be affected by varying the basal transcription rate (α_b_) and the strength of the Tat-mediated positive feedback (c) (see **Methods** for a description of these parameters). We simulated HIV promoter activity up to 10 days and compared how heterogeneous distributions in endpoint protein values changed for different values of basal transcription and positive feedback strength (Fig. 1C). As basal transcription is increased from 0.01 to 10 day^−1^, mean counts of Tat steadily increase for every value of positive feedback strength analyzed (from 0.01 to 100). In particular, for all positive feedback strengths sampled, the distributions transition through three stages characterized by increasing means (μ) of Tat production: an ‘initial’ state (μ < 1), an ‘intermediate’ state (μ > 1, although some cells still have Tat counts of 0), and a ‘final’ state (μ > 1 and all cells have Tat > 0). We note that for the lowest feedback strength (c = 0.01), the endpoint protein distributions are similar to a model with no Tat positive feedback (Fig. 1C, **far right**).

To quantify how varying basal transcription rate and positive feedback strength affects noise in gene expression, we computed Fano factor (defined as variance over mean; ρ = σ^2^/μ; units of protein counts) and coefficient of variation squared (CV^2^; defined as variance over mean-squared; σ^2^/μ^2^) for the endpoint protein distributions. Our analysis shows that for basal transcription rates greater than 0.1 day^−1^, the Fano factor rises and falls as the strength of the positive feedback increases. Moreover, as the basal transcription rate decreases, peaks in Fano factor occur at higher feedback strengths (Fig. 1D). For basal transcription rates of 0.1, 0.5, and 1 day^−1^, the Fano factor peak corresponds to the system in an ‘intermediate’ phenotype (μ > 1, Tat ≥ 0) (Fig. 1C**, red, blue, and magenta asterisks**). However, for a basal transcription rate of 10 day^−1^, the Fano factor peak is associated with a high productivity state across all cells (μ > 1, Tat > 0) (Fig. 1C**, green asterisk**). The trend in Fano factors in the one-state model suggests that for high basal transcription rates, noise profiles peak when transcription is weakly amplified by feedback, causing a widening of the endpoint protein distribution. However, for low basal transcription rates, noise profiles peak when transcription is amplified by strong feedback, and cell populations sample an ‘intermediate’ phenotype that may correspond to different cell fates (i.e., the presence and absence of protein product). Meanwhile, CV^2^ decreases monotonically as basal transcription is increased (Supplementary Figure S1). Notably, as positive feedback is strengthened and cells transition across ‘initial’ (μ < 1) or ‘intermediate’ (μ > 1, Tat ≥ 0) states to higher productivity phenotypes (μ > 1, Tat > 0), CV^2^ drops (as can be seen in trend lines for basal transcription rates of 0.1, 0.5, and 1 day^−1^; Supplementary Figure S1).

Next, we calculated mean onset time under varying conditions of basal transcription and strength of the positive feedback. We defined onset time as the earliest presence of non-zero protein counts within a time trace. If protein counts were zero for the entire course of the simulation, mean onset time was set artificially as 10 days–the maximum amount of the time that the simulations were allowed to run. The strength of the positive feedback did not affect mean onset time, while the basal rate of transcription did (Fig. 1E). As the basal transcription rate was increased, the mean onset time decreased approximately linearly. Note that at lower basal transcription rates, the inverse relationship to mean onset time appears non-linear due to the upper limit of 10 days.

The sensitivity analysis of a one-state model with positive feedback demonstrated that we could simulate a system that exhibited distinct stages of Tat production under varying basal transcription rates. At a positive feedback strength of 1, the system clearly exhibits three distinct phases as basal transcription is increased–an ‘initial’ state with low Fano factor, an ‘intermediate’ state with increased Fano factor, and a ‘final’ state with low Fano factor and high mean (Fig. 1C). Therefore, for our remaining simulations, we set positive feedback strength to 1 in order to deemphasize Tat regulation within the cell (see **Methods**), and rather focus on assessing noise within the viral genetic circuit due to promoter activation mechanisms, transcription, and translation.

Furthermore, we used the one-state model to set a threshold for viral activation, occurring between ‘intermediate’ and ‘final’ states of Tat production (i.e., between basal transcription rates of 1 and 10 day^−1^ at a feedback strength of 1 from Fig. 1C), beyond which we assumed cells would be associated with a fully productive infection (or “ON” state). Therefore, we set basal transcription rate to 3 day^−1^, which resulted in a one-state model that exhibited monostable behavior with a stable equilibrium point at approximately 316 Tat protein molecules (see **Methods** and Supplementary Figures S2). In subsequent analyses, we used this threshold to explore how the dynamics and noise profiles of two- and three-state promoter models affect heterogeneity of viral activation phenotypes.

### Cooperativity and transcriptional amplification increase noise in the one-state model

We next explored how adding cooperativity to Tat positive feedback would affect dynamics and noise profiles in the one-state model (Supplementary Figure S3A). Biologically, cooperative feedback describes a mechanism by which multiple copies of Tat are necessary to auto-stimulate Tat expression. The experimental literature demonstrating Tat cooperativity is limited. While a few studies show that Tat can dimerize^23^, or multiple Tat molecules interact with the RNAPII-TAR complex during transcription^24^, most have argued against a cooperative mechanism^25^,^26^. For this reason, previous computational studies have generally modeled Tat positive feedback without requiring cooperativity; however, we include it here to consider how it affects activation noise.

The Hill coefficient (q) was varied from 1 to 5 at a basal transcription rate of 3 day^−1^. When multiple steady states were present (e.g., for q = 3), the simulation runs tended to stabilize at the lower steady state value because the model was run with an initial Tat protein count of 0 (Supplementary Figure S3B). When compared to a model without cooperative feedback at the same basal transcription rate (Supplementary Figures S2), the cooperative feedback model (e.g., for q = 3) exhibited bistable behavior (indicative of two stable phenotypes) with greater tendency towards the unproductive state (only 103 out of 1000 productive simulations; Supplementary Figure S3C). Overall, cell activation decreased with increasing q, while Fano factor peaked at a q of 3 (Supplementary Figure S3D). Mean onset time and mean first passage time did not change significantly with increasing q.

Finally, in modeling the one-state system, we had assumed a production of one transcript per event. However, previous studies have demonstrated that transcriptional amplification may play a role in increasing gene expression noise^27^,^28^. Therefore, we assessed the influence of transcriptional amplification (AMP) of 10 and 100 on noise profiles in the one-state promoter model wherein multiple transcripts are synthesized per event (see **Methods** and Supplementary Figure S4A). When basal transcription was again set at a rate of 3 day^−1^, cell activation was already close to 100%, but variability in the protein levels of activated cells increased greatly with strong amplification leading to very large differences in Fano factor (Supplementary Figure S4B).

### Assessing metrics of viral activation and noise across varying transcriptional bursting behaviors in a two-state promoter system

**Figure 2:**
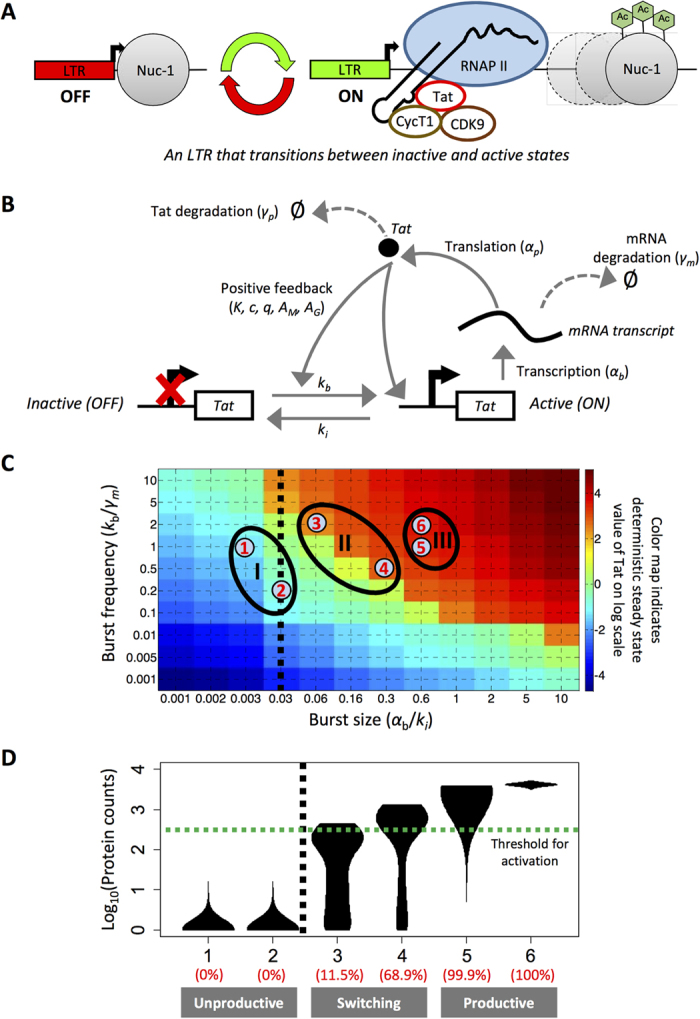
Conceptual schematic and transcriptional bursting behaviors in a two-state promoter model with positive feedback. **(A)** Conceptual diagram of the underlying biological mechanisms that define inactive and active LTR states. **(B)** Schematic depiction of a two-state computational model with positive feedback. **(C)** Heat map of the deterministic steady-state Tat protein levels for the two-state model with positive feedback simulated over a range of burst sizes and burst frequencies (presented in log scale). Burst size is computed as the Tat-independent transcription rate divided by the promoter inactivation rate (α_b_/k_i_). Burst frequency is computed as the promoter activation rate divided by the transcript degradation rate (k_b_/γ_m_). The color map indicates the deterministic steady state value of Tat (in log scale). The black dotted line at a burst size of 0.03 is equivalent to the Tat-mediated transcription rate used in the active one-state model with positive feedback. Three distinct regions are depicted on the color map (as I, II, and III), and these correspond to regions of unproductive, switching, or productive behavior, respectively. **(D)** Violin plots of the steady-state protein distributions for the corresponding parameter sets of burst size and burst frequency in **(C)**. The green dotted line indicates the threshold for activation (Supplementary Figure S2). The black dotted line is the same as in **(C)**. Numbers in red indicate cell activation percentages.

In most eukaryotic cells, transcription occurs in bursts as a gene transitions infrequently from an inactive to an active state, thus yielding large cell-to-cell variation in mRNA molecules^4^,^9^,^29^. Experimental evidence suggests that nucleosome remodeling contributes to transcriptional bursting behavior. Promoters are often silenced in the presence of a nucleosome (i.e., produce few to no transcripts), but can drive high levels of transcription once the nucleosome is displaced or repositioned. Others have speculated that the requirement for transcription factor-mediated recruitment of multi-component transcriptional complexes could be an underlying cause of transcriptional bursting^30^,^31^. In the specific case of the HIV LTR promoter, restrictive Nuc-1 is present immediately downstream of the start site, occluding transcription, and thus needs to be displaced by chromatin remodeling complex PBAF^16^,^22^. Moreover, transcription factors NF-κB, NFAT, and Sp1 all play a critical role in transcription initiation and promoter clearance^16^. For our purposes, we assumed the influence of chromatin and cis-acting regulatory elements were represented by two distinct promoter states (Fig. 2A).

Mathematically, activation from distinct promoter states is represented by probabilistic transitions between inactive and active states (referred to as a ‘two-state model’; Fig. 2B). The bursting behavior of the model is defined by two parameter groups: burst size and normalized burst frequency. Burst size, or the number of transcripts produced when the promoter is in the active state, is defined as the Tat-independent transcription rate (α_b_) divided by the inactivation rate of the promoter (k_i_). Burst frequency is defined as promoter activation rate (k_b_) normalized to the transcript degradation rate (γ_m_). We further assumed that Tat positive feedback increases both α_b_ and k_b_.

Two-state models have been used previously to model HIV transcription^8^,^30^,^32^,^33^ because constitutive, one-state models of gene expression were unsatisfactory in explaining noise in HIV gene expression. For example, modulation of burst size and burst frequency parameters in a two-state model was able to accurately describe experimentally observed gene expression variability for LTRs integrated across the genome^32^,^33^. Notably, these experimental systems and computational models lacked Tat-positive feedback. A subsequent study incorporated Tat-positive feedback with a two-state gene model to explore the influence of mutations at the Sp1 binding site within the HIV LTR on heterogeneous phenotypes^19^.

Here, we investigated how the greater parameter space afforded by the addition of an inactive state would increase the range of heterogeneous reactivation behaviors when coupled to feedback. To this end, we solved the steady-state deterministic solutions for different parameter sets of burst size and frequency (Fig. 2C). Then, we identified three parameter regions of interest by simulating two different parameter sets within each region and solved for the end-point protein distributions using a stochastic modeling approach (Fig. 2D). We categorized these regions as: fully unproductive (all cells have Tat < threshold), variably productive (some cells have Tat > threshold), and fully productive (all cells have Tat > threshold). Similar to the results for the one-state model, as the basal transcription rate is increased either by increasing burst size or burst frequency, the cell populations transition from unproductive unimodal states to variably productive, typically bimodal states, and then to fully productive unimodal states.

**Figure 3:**
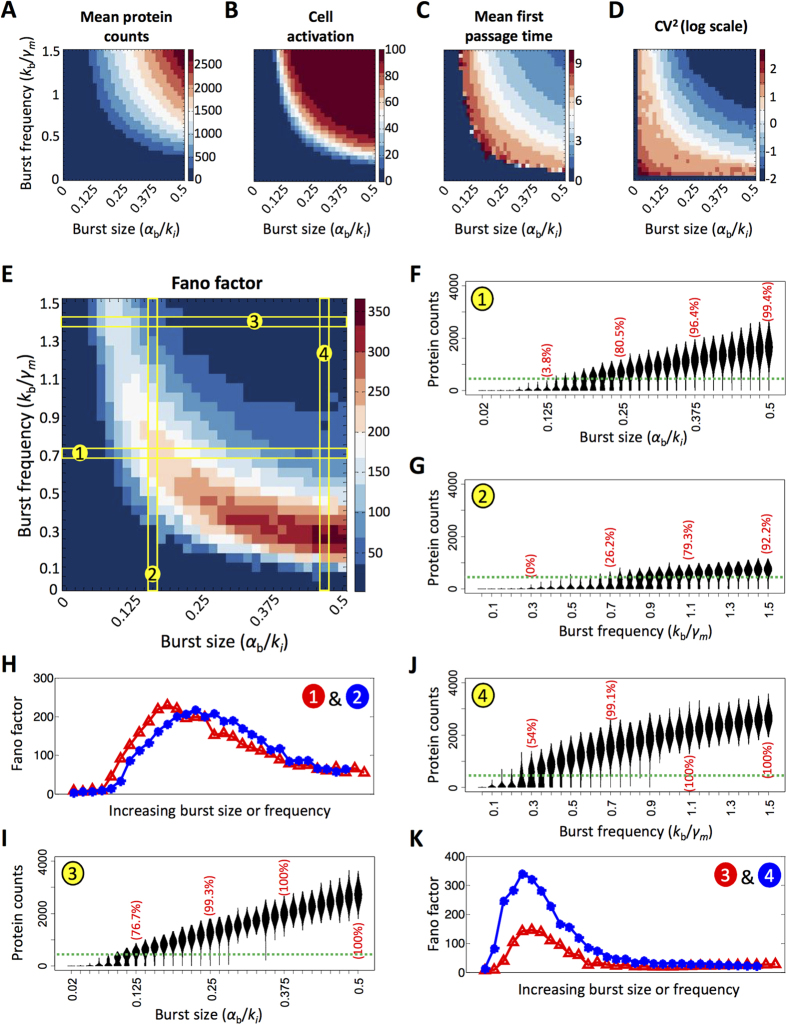
Assessing system heterogeneity under varying transcriptional burst sizes and frequencies for the two-state promoter model. **(A–E)** A two-state model with positive feedback was simulated for a range of burst sizes and burst frequencies and the following metrics were calculated based on the final Tat protein values: **(A)** Mean protein counts; **(B)** cell activation; **(C)** mean first passage time (days); **(D)** coefficient of variation squared (presented in log scale); and **(E)** Fano factor. Color bars in each panel indicate the range of values for each metric. **(F,G,I,J)** Violin plots capturing endpoint protein distributions across paths charted in **(E). (H,K)** Plots of Fano factor across increasing burst size or frequency correspond to paths charted in **(E)**.

Focusing specifically on the region characterized by variably productive states, we varied burst size and burst frequency and computed mean protein counts (Fig. 3A), cell activation (Fig. 3B), and mean first passage time (i.e., the minimum amount of time it takes for a cell to transition from an unproductive to a productive state) (Fig. 3C), as well as mean onset time (Supplementary Figure S5). As burst size and frequency was increased, mean protein counts increased as expected (Fig. 3A). Moreover, the bands of varying mean protein counts are roughly symmetrical along the diagonal indicating that very high burst frequency coupled with low burst size or very high burst size coupled with low burst frequency will result in similar means. At the band indicating protein counts between 250 and 500, a small fraction of cells begin to occupy the activated state, as determined by the threshold of ~316 Tat proteins (Fig. 3B). However, on average these cells tend to have a very late first passage time (Fig. 3C). The heat maps for cell activation and first passage time together demonstrate the minimum burst size and frequency necessary to activate transcription in the two-state model, and a full transition from an unproductive to productive cell population occurs within a relatively narrow parameter region. We note that even for populations with fully productive end points, characterized by relatively high burst sizes and burst frequencies, wide variations in first passage time exist.

For the two-state model, we examined different measures of noise: the square of the coefficient of variation or CV^2^ (σ^2^/μ^2^; Fig. 3D), and Fano factor (σ^2^/μ; Fig. 3E) of endpoint protein distributions. Interestingly, these measures of noise peak in different phenotypic regions. We observe that CV^2^ peaks at low burst frequency and low burst size, when mean protein counts are very low (Fig. 3D). For any fixed burst size, CV^2^ decreases monotonically as burst frequency increases, similar to the observation for mean onset time (Supplementary Figure S5). Likewise, for any fixed burst frequency, CV^2^ decreases as burst size increases, except at the very low (non-zero) burst frequencies. In contrast, Fano factor is low at low values of burst size and burst frequency and rather peaks at the highest burst sizes paired with the lower range of burst frequencies sampled (Fig. 3E). The Fano factor is driven largely by burst size in a two-state model without feedback^34^; however, in the presence of feedback, it is also dependent on the burst frequency. In contrast to CV^2^, which reaches a minimum level as the cell population becomes fully productive, the Fano factor peaks as the cell population moves through a region of variably productive phenotypes, and decreases as the cells become fully productive. The highest Fano factors are thus associated with intermediate levels of cell activation, suggesting that the Fano factor is a more informative measure of phenotypic heterogeneity in this system.

To better understand how Fano factor is coupled to phenotypic heterogeneity, we mapped paths in parameter space that transitioned the population from 0% to >90% cell activation (i.e., from fully unproductive to variably/fully productive), and calculated the corresponding Fano factor ranges. In the first case, we examined the behavior of cell populations that transitioned over similar Fano factor ranges but displayed distinct heterogeneous phenotypes. Burst frequency was held constant at 0.7 while burst size was increased (Fig. 3E, **path 1** and Fig. 3F), or burst size was held constant at 0.16 while burst frequency was increased (Fig. 3E**, path 2** and Fig. 3G). While the Fano factor profiles of paths 1 and 2 appeared similar (Fig. 3H), transitioning the population by increasing burst size resulted in bimodal phenotypes for a narrow range of burst sizes, but was associated with a widening “ON” peak and high maximum protein counts (Fig. 3F). By contrast, when cells were transitioned by increasing burst frequency, cells exhibited distinct bimodal phenotypes over a wide range of burst frequencies, but the maximum protein count was much lower and the width of “ON” peak plateaued (Fig. 3G). In the second case, cell populations were transitioned from low to high productivity but with low or high Fano factor peaks. In this case, burst frequency was held constant at 1.4 while burst size was increased (Fig. 3E**, path 3** and Fig. 3I), or burst size was held constant at 0.47 while burst frequency was varied (Fig. 3E**, path 4** and Fig. 3J). For these specific paths, both transitions occur over a narrow parameter range with similar endpoint protein distributions (Fig. 3I,J), but the Fano factor peaks are distinct (Fig. 3K). Path 4 has a high Fano factor peak associated with a region of distinct bimodal populations (Fig. 3J), while Path 3 has a lower Fano factor peak associated with less distinct bifurcations (Fig. 3I). Interestingly, if the same sets of paths are charted in a plot of coefficient of variation (σ/μ), these noise profiles appear nearly identical (Supplementary Figure S6). Thus, Fano factor is a good indicator of heterogeneous phenotypes, with the highest values generally associated with bimodal distributions.

Fano factor values are also associated with patterns in first passage time. Examining first passage time for paths 1 and 3, we see that at higher burst frequencies, the transition from an unproductive to productive state is faster than at lower burst frequencies. Correspondingly, higher burst frequencies are associated with decreased Fano factor. Meanwhile at lower burst frequencies, cells are delayed in the process of transitioning to a productive state, and therefore, they are associated with the highest Fano factors at increasing burst size values.

Taken together, these observations suggest that “tuning” burst size and burst frequency in the two-state model with feedback has differential effects on the distribution of protein counts. For instance, increasing the burst frequency at low, constant burst sizes reduces the variability in protein count in “ON” cells. By contrast, increasing burst size raises the mean protein count in “ON” cells by extending the distribution tail length, which results in increased variability.

### Assessing the effect of Tat compartmentalization on noise and viral activation behaviors in a two-state promoter system

We also considered the influences of cellular compartmentalization on viral activation and noise in order to understand if additional steps in Tat production and positive feedback might influence the overall behavior of the system. First, we built a compartmentalized version of the two-state model by introducing additional reactions (see **Methods**). Mainly, an export reaction for mRNA from the nucleus to cytoplasm and an import reaction for Tat from the cytoplasm to the nucleus were both added (Supplementary Figure S7A). We further replaced generic mRNA and Tat species with nuclear mRNA (mRNA_n_), nuclear Tat (Tat_n_), cytoplasmic mRNA (mRNA_c_), cytoplasmic Tat (Tat_c_), and modified the reaction propensities such that only nuclear Tat_n_ affected positive feedback. At physiological rates of mRNA export and Tat import chosen from previous modeling studies (see **Methods** for rates), we observed that metrics for viral activation and noise (mean first passage time, cell activation, Fano factor, and mean onset time) did not change significantly from a two-state model without compartmentalization (compare Supplementary Figure S7B with Fig. 3). Only when the transport rates were slowed 10 to 100-fold did we observe significant variations in viral activation and noise, including significant delays in onset time of Tat production (Supplementary Figure S7C and S7D). Therefore, we conclude that promoter bursting contributes more to noise in HIV reactivation than Tat compartmentalization.

### Transcriptional bursting behaviors in a three-state promoter system and comparison of noise profiles with a two-state promoter system

**Figure 4:**
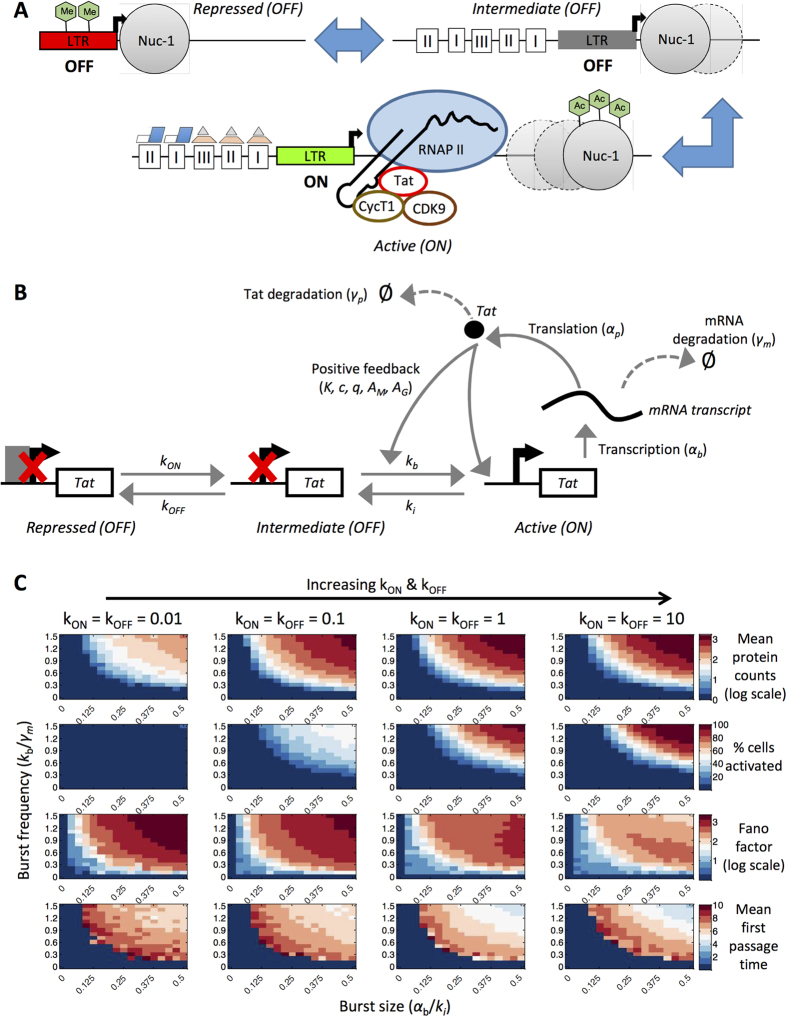
Conceptual schematic and transcriptional bursting behaviors in a three-state promoter model with positive feedback. **(A)** Conceptual diagram of possible biological mechanisms underlying three promoter states (two inactive and one active). **(B)** Schematic depiction of a three-state computational model with positive feedback. **(C)** Three-state models with positive feedback were fixed at different values of k_ON_ and k_OFF_ and then simulated over a range of burst sizes and burst frequencies. The following metrics were calculated based on the final Tat protein values: mean protein counts (in log scale), cell activation, Fano factor (in log scale), and mean first passage time (days). Color bars in each panel indicate the range of values for each metric.

Although two-state models have been successfully used to describe experimental observations of transcription from the HIV LTR promoter^32^,^33^, in all cases these promoters are more productive (with higher burst sizes) than we expect for latent viral integrations *in vivo*. In another experimental study of transcriptional bursting across the mammalian genome it was observed that the time spent in the inactive promoter state can be very long^35^. In these cases, the inactive state is better described by a refractory period in the “OFF” state modeled by two sequential processes, before the gene can be switched on again^36^. Including multiple inactive states via a three-state promoter system could provide a means to model a highly repressed stage characterized by hypermethylated CpG islands, which are considered distinct from the more reversible form of silencing mediated by the recruitment of histone deacetylases^16^ (Fig. 4A).

To account for the presence of different silencing mechanisms, we described the three-state model with two inactive states: repressed “OFF” and intermediate “OFF” states. We considered transitions from the repressed to intermediate state (e.g., indicative of DNA methylation reversal) and from the intermediate to active state (e.g., indicative of acetylation; Fig. 4B). Tat-mediated positive feedback amplified the transcription rate in the active state (α_b_) and the transition rate from the intermediate inactive state to the active state (k_b_), but it did not affect the transition from the repressed to intermediate “OFF” state, consistent with our biological assumptions.

We varied the parameters accounting for the transition between the repressed and intermediate states (k_ON_ and k_OFF_) over four orders of magnitude (0.01, 0.1, 1, and 10 day^−1^). This range for k_ON_ allowed us to sample rates that are smaller or on the same order of magnitude as k_b_ (0.48 to 7.2 day^−1^ for the three-state model). For k_OFF_, the range of 0.01 to 10 day^−1^ is slower than the k_i_ value fixed in the model (96 day^−1^), based on the assumption that these longer-term remodeling events represented by the transition from the intermediate to repressed state would occur more slowly than the transition from the active to intermediate state. We again varied transcriptional burst size and frequency as in the two-state model and computed mean protein counts, cell activation, Fano factor, and mean first passage time (Fig. 4C and Supplementary Figures S8–S11). We observed that k_ON_ and k_OFF_ rates of 0.01 day^−1^ are not enough to activate cells by the end of ten days. A small fraction of cells activate at k_ON_ and k_OFF_ rates of 0.1 day^−1^ (Fig. 4C). Adjusting the rates of k_ON_ and k_OFF_ relative to each other produced expected results. For instance, increasing k_ON_ while holding k_OFF_ constant produced greater cell activation and trends in Fano factor began to mimic the two-state model more closely. However, increasing k_OFF_ while holding k_ON_ constant decreased cell activation leading to unproductive simulations (Supplementary Figures S9 and S10).

**Figure 5:**
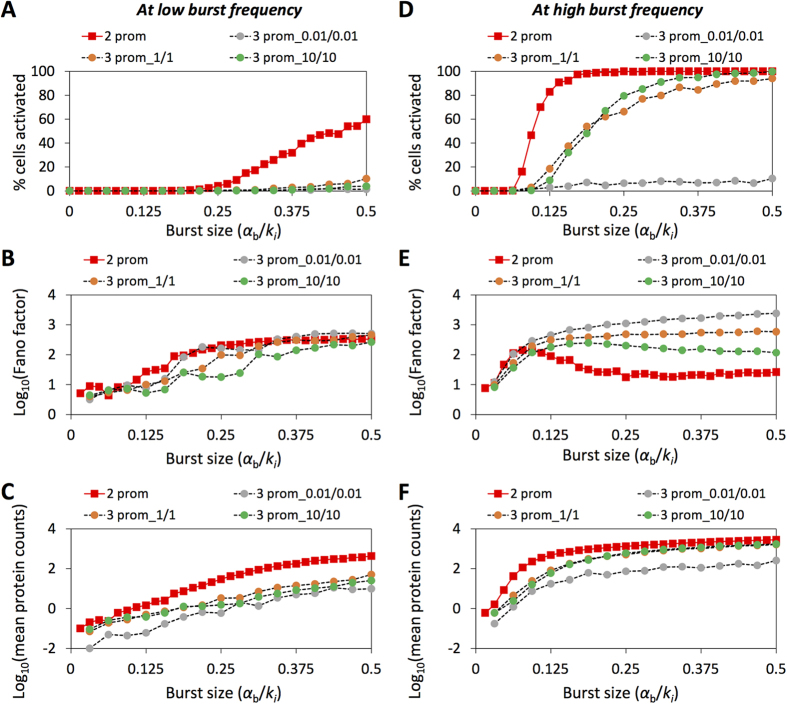
Distinctive activation and noise profiles at low and high burst frequency for two- and three-state promoter models with positive feedback. **(A–C)** Two- and three-state models were fixed at a low value of burst frequency (0.3) and then simulations were run for a range of burst sizes. Final Tat protein values were used to calculate **(A)** cell activation, **(B)** Fano factor (in log scale), and **(C)** mean protein counts (in log scale). **(D–F)** Same as in **(A–C)** but burst frequency was set to a high value (1.5). The three-state model was simulated with k_ON_ = k_OFF_ = 0.01 day^−1^ (‘3 prom_0.01/0.01’), k_ON_ = k_OFF_ = 1 day^−1^ (‘3 prom_1/1’), and k_ON_ = k_OFF_ = 10 day^−1^ (‘3 prom_10/10’). Two-state model is labeled as ‘2 prom’.

Observing transition rates from k_ON_ = k_OFF_ = 0.01 up to k_ON_ = k_OFF_ = 10 day^−1^, relative Fano factor values provide an indication of the most important changes in cell activation dynamics as compared to a two-state model, but these differences depend on the value of burst frequency for the transition to the active state (Fig. 5). At a low burst frequency value of 0.3, Fano factor increases with burst size in both the two and three-state models; however, while the two-state model achieves intermediate cell activation (60%), the three-state models analyzed achieve no more than 11% activation (Fig. 5A,B). Note that for the three-state models, mean protein counts are generally less than the activation threshold for this burst frequency value (Fig. 5C). At a high burst frequency value of 1.5, the Fano factor trends for the three-state model change significantly for different values of k_ON_ and k_OFF_. For k_ON_ = k_OFF_ = 10, the Fano factor peaks and begins to gradually fall as the cells transition from a fully unproductive to a fully productive state (Fig. 5D,E). In contrast, a three-state model with k_ON_ = k_OFF_ = 0.01 is associated with a very high and increasing Fano factor and a low percentage of cells activating over the analyzed burst size range. At a high burst frequency value, raising k_ON_ from 0.01 to 10 results in a corresponding increase in mean protein counts from below to above the activation threshold (Fig. 5F and Supplementary Figure S8). Overall, Fano factor proves to be a useful metric to compare and distinguish heterogeneous population behaviors between the two- and three-state models.

### Experimental measurements of HIV protein distributions are qualitatively described by a three-state model

**Figure 6:**
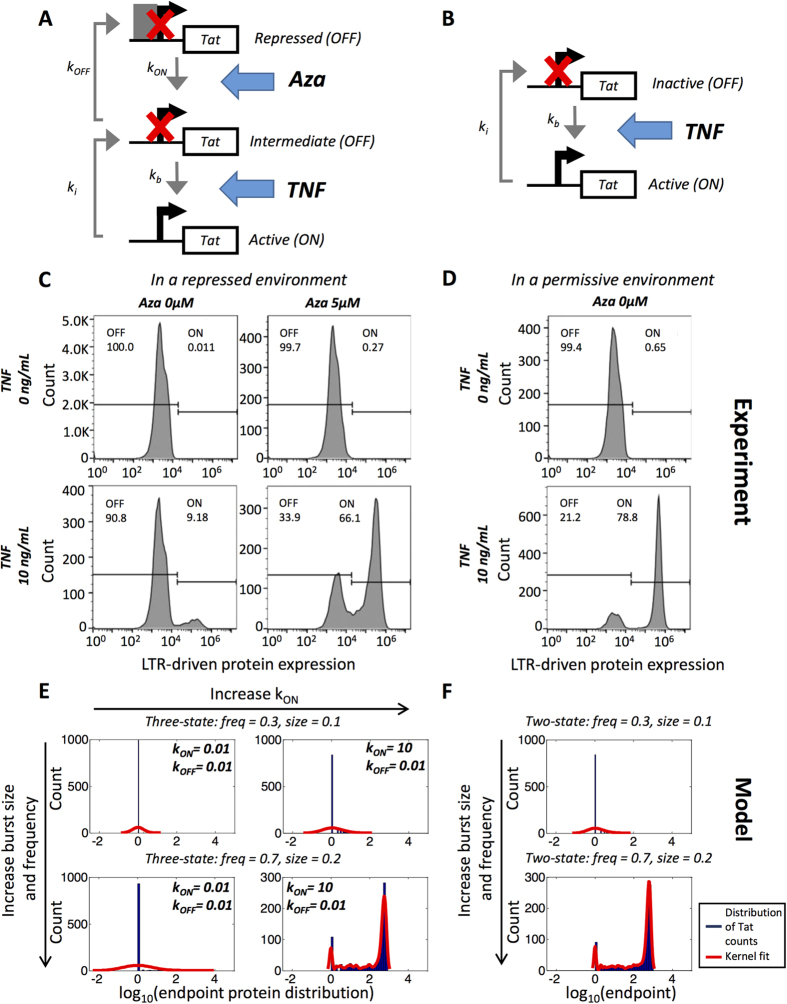
Comparing sample experimental and simulated latent HIV infections under basal and stimulated conditions. **(A,B)** Conceptual diagrams illustrating the action of Aza and TNF on **(A)** three-state and **(B)** two-state models. **(C,D)** Flow cytometry histograms of GFP expression for two HIV latency cell line models that are **(C)** repressed or **(D)** permissive for activation by TNF. Histograms are shown under basal conditions and following stimulation with 10 ng/ml TNF and/or 5 μM Aza. **(E)** Three-state model simulation of the experimental data presented in **(C)**. TNF is assumed to increase burst size and burst frequency and Aza is assumed to increase the transition between inactive states (k_ON_). **(F)** Two-state model simulation of the experimental data presented in **(D)**.

The three-state model of the LTR promoter can be used to more accurately describe experimental latency cell line models that have multiple mechanisms of repression, which may in turn require different combinations of drugs to reverse latency. For example, the additional parameter space afforded by a third promoter state can be used to describe highly restrictive epigenetic modifications such as CpG methylation that are characteristic of some *in vitro* HIV latency cell lines^37^, but are not present in others (Fig. 6A versus 6B). Importantly, it can be difficult to detect differences in chromatin repression across different HIV latency cell lines simply by measuring experimental protein distributions in the basal state by flow cytometry. For example, the basal protein expression for an HIV latency cell line in which transcription is highly repressed by CpG methylation (Fig. 6C, **top left**) is virtually identical to the basal protein expression for a cell line in which there is relatively little CpG methylation, but hypoacetylation, and low transcription factor levels that, when combined, may only be weakly maintaining the latent state (Fig. 6D**, top**). However, when these different cell lines are stimulated with drugs to reverse latency, the differences in repression lead to clear differences in response. For example, CpG-methylated LTRs exhibit little expression after tumor necrosis factor (TNF) stimulation (Fig. 6C**, bottom left**), while permissive LTRs exhibit strong expression after stimulation (Fig. 6D**, bottom**). Reversing CpG methylation via chemical perturbation with 5-aza-2-deoxycytidine (Aza), a CpG methylase inhibitor, does not increase expression on its own (Fig. 6C**, top right**). However, co-stimulation of the CpG-methylated promoter with TNF and Aza results in expression that is similar to the permissive HIV latency model cell line (Fig. 6C**, bottom right**).

A computational model with three promoter states provides the necessary parameters to describe these differences in repressive mechanisms acting at the promoter and can qualitatively capture the effect of drug combinations. For example, TNF causes acetylation of histones and increased transcription factor binding at the promoter^38^, which we model as an increase in both burst size and burst frequency (Fig. 6E,F). Aza reverses CpG methylation^39^, which we model as an increase in k_ON_. By increasing only burst size and burst frequency while keeping k_ON_ constant (i.e., adding only TNF) or by increasing only k_ON_ while keeping burst size and burst frequency constant (i.e., adding only Aza), our model accurately predicts there will be no increase in expression (Fig. 6E). However, simulating the addition of both drugs by increasing all parameters results in substantial synergistic activation (Fig. 6E**, bottom right**). In contrast, a two-state model is sufficient to capture experimental observations for a non-CpG-methylated promoter (Fig. 6F). We note that this more permissive behavior could be captured with a three-state model by assuming high initial values of k_ON_. Overall, we conclude that three-state promoter models may provide more accurate representations of viral latency that results from multiple biological regulatory mechanisms and exhibits complex responses to drug perturbation.

## Conclusions

Previous efforts to simulate HIV transcription and latency via mathematical models have assumed either one or two promoter states, with some studies including Tat-positive feedback and others choosing to exclude it^17^,^19^,^30^,^32^,^33^,^40^,^41^. In this article, we took a more comprehensive approach and present a comparative analysis of multiple promoter activation mechanisms featuring many feedback structures and variations in transcriptional bursting behaviors. We also chose to explore parameter space encompassing very low basal transcription rates that was not considered in previous studies but may be more representative of latent viral behavior. Furthermore, we presented the novel use of a three-state model (consisting of two inactive states and an active state) in the specific context of HIV and combined with Tat-positive feedback. We find that this model more accurately reflects observed heterogeneous reactivation, and thus may be better suited to evaluate how noise affects viral clearance.

While earlier studies have fit experimental chemical perturbation data derived from cell-line HIV latency models to a two-state LTR model^8^,^30^,^31^,^32^,^33^, our results suggest that the addition of a third state provides more parameters with which to describe the mechanisms that maintain latency and may more accurately fit experimental data. Two classes of compounds–activators of transcription factors and histone deacetylase (HDAC) inhibitors–are promising examples of HIV latency reversing agents (LRA) that have been tested across multiple latency models and in patient samples^42^,^43^,^44^. However, these drugs often do not result in complete activation, even in cell line latency models, in part due to additional mechanisms of repression, including CpG methylation and histone methylation^37^,^45^. The three-state model provides a means to mathematically describe these additional repressive states and more effectively simulate how combinatorial treatments will affect integration sites that are highly restrictive to transcription.

Ultimately, the modeling frameworks presented in this article allowed us to explore how stochastic fluctuations at the level of the promoter contribute to experimentally observed cell-to-cell phenotypic variability in reactivation from latency. Even though simple computational models as presented in this article can recapitulate a tremendous amount of complexity present in experimental observations, future modeling efforts can center on more detailed and accurate representations of chromatin biology and transcriptional regulation.

## Methods

### The one-state model of HIV LTR promoter with Tat-positive feedback

The reactions incorporated in the one-state model with feedback (Fig. 1B) are as follows:


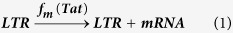



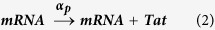



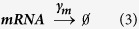



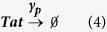


A deterministic model of the one-state system with feedback can be represented by the following differential equations:


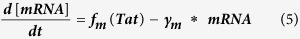



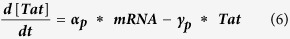


Here, LTR refers to the HIV promoter which is always in the ‘active’ state; 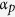 is the mRNA translation rate; 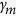 is the mRNA degradation rate; and 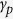 is Tat degradation rate. The function 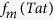 incorporates mRNA transcription from the HIV LTR as well as Tat transactivation and is given in a Hill form:


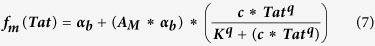


In **equation** 7, 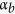 is the basal rate of mRNA transcription, 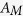 is the Tat-mediated amplification factor for mRNA transcription, *K* is the effector concentration for half-maximum response for the feedback as a function of Tat, *c* is the strength of the positive feedback, and *q* denotes the Hill co-efficient.

The basal HIV transcription rate is the rate of production of viral transcripts in the absence of Tat transactivation. HIV integrates semi-randomly into the genome^46^, and therefore, the basal rate of transcription can vary widely depending on the local chromatin environment of the LTR promoter^47^. Once Tat positive feedback is initiated, the basal rate of transcription is amplified to account for the Tat-mediated transactivation of transcription. The strength of the Tat-mediated positive feedback describes how much each Tat molecule contributes to transactivation, which can be affected by the presence of Tat regulators within a cell^18^,^26^,^48^. Here, we assume the level of amplification (A_M_) provided by Tat activity is constant across all integration sites, and Tat transactivation follows a Hill form with characteristic constant ‘K’ and a Hill co-efficient ‘q’. For the above model, the chosen parameters are listed in Table 1.

The deterministic equations ignore fluctuations in the system with regard to mRNA and Tat counts. Therefore, to capture the effects of these fluctuations, we employ a stochastic modeling approach. But first, we record the reaction propensity (ν) and reaction stoichiometry (S). The reaction propensity tells us how frequently a reaction occurs while the stoichiometry tells us how much the system is changed when the reaction is completed.

For synthesis:


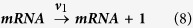



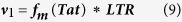



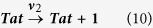



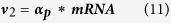


For degradation:


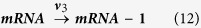



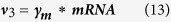



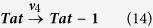



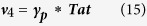


Therefore:


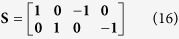



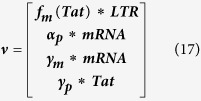


Because the LTR is always active in the one-state model, it remains unchanged.

### Determining an activation threshold using the one-state model

Previous experimental observations have demonstrated that cells with low and high levels of Tat expression can co-exist within clonal populations and that high Tat expression can result in ‘productive’ viral replication, while low Tat expression allows cells to remain ‘unproductive’^17^. In our simulations of the one-state model, the three distinct stages of Tat production (‘initial’, ‘intermediate’, and ‘final’) were characteristic of cell populations with variegated expression profiles. However, we needed to establish an activation threshold, occurring between ‘intermediate’ and ‘final’ states of Tat production, beyond which cells would always be associated with fully productive infection. To set the activation threshold, the basal transcription rate for the one-state model was fixed at 3 day^−1^, which resulted in monostable behavior indicative of a fully productive infection (Supplementary Figure S2). When solved deterministically, the system had a stable equilibrium point at approximately 316 Tat protein molecules, and when implemented stochastically, 962 out of 1000 simulations crossed this threshold at least once during the time trace (see example of “ON” trace in Supplementary Figure S2).

### Modeling transcriptional amplification using the one-state model

To model transcriptional amplification, mRNA synthesis was modeled as:


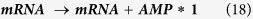


Which altered the stoichiometric matrix as follows:


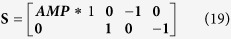


### Extension to a two-state model of the HIV LTR promoter with positive feedback

The additional reactions incorporated in a two-state model (Fig. 2B) are as follows:


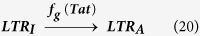



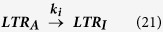


The reactions describing transcript and protein production/degradation (**equations** 1 through 4, 2, 3, 4) remain the same. In **equations** 20 and 21, LTR_I_ refers to an ‘inactive’ HIV promoter in the “OFF” state, and LTR_A_ refers to an ‘active’ promoter in the “ON” state. And, the function 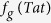, similar to (**equation** 7), incorporates Tat influence on gene activation as follows:


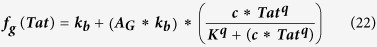


In **equation** 22, *k*_*b*_ is the rate of gene activation, and *A*_*G*_ is the Tat-mediated amplification factor. Below, we record the reaction propensity (ν) and reaction stoichiometry (S) for the two-state model.

Promoter transition states; assume LTR = 1 for active state (LTR_A_) and LTR = 0 for inactive state (LTR_I_):


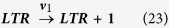



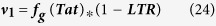



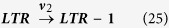



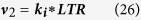


The reactions describing mRNA and Tat synthesis (**equations** 8 through 15, 15, 45, 46, 47, 39, 40, 41) remain the same. Therefore, the stoichiometric matrix and reaction propensity vector for the two-state model are as follows:


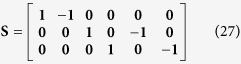



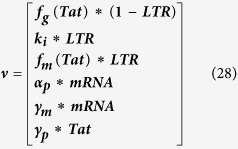


For the above model, the chosen parameters are listed in Table 1.

As seen in the two-state model chosen, Tat positive feedback is modeled to influence both the gene activation rate (k_b_) as well as the promoter transcription rate (α_b_). To better understand the contributions of Tat positive feedback mechanisms to the overall behavior of the two-state model, we ran simulations wherein one of the feedback loops was knocked out each time. The results indicate that the feedback loop which directly affects the transcription rate from the promoter (as opposed to the gene activation rate) is particularly sensitive across variations in transcriptional burst size and frequency (see Supplementary Figure S12). The feedback loop affecting the gene activation rate does not by itself lead to productive cell behavior at the sampled transcriptional burst sizes and frequencies.

### Modeling the effect of Tat compartmentalization using a two-state promoter model

The following additional reactions were incorporated into the two-state model:


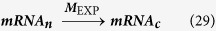



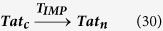



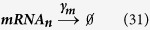



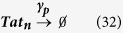



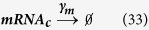



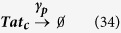


Here, *M*_*EXP*_ refers to the mRNA export rate of 62.2 day^−1^ 49 and *T*_*IMP*_ refers to Tat import rate of 499.68 day^−1^ 50. Furthermore, both mRNA and Tat are separated into their nuclear (mRNA_n_ and Tat_n_) and cytoplasmic (mRNA_c_ and Tat_c_) components. The rates for transcript and protein degradation (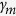 and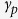) remain the same as before. Additionally, the two-state model was modified such that only Tat_n_ participated in the transactivation process.

### Experimentally measured ranges of transcriptional burst sizes and frequencies

The bimodal state is indicative of a variegated expression phenotype wherein cells with low and high mean Tat levels can co-exist within a population. Interestingly, we find that this region of interest occurs within a range of burst frequencies (0.1 to 1.5) that matched those fit from experimental data in previous studies of the LTR^8^,^33^. In contrast, the burst size range was at least an order of magnitude lower than the ranges measured previously (<0.5) (Supplementary Table S1). Burst sizes extending into experimentally measured ranges all produced fully productive cell populations in our simulations with strong amplification by Tat-mediated positive feedback (Supplementary Figure S13). We hypothesize that the previous experiments, which used HIV LTR-driven GFP reporters that lacked Tat, were unable to identify cells with these more repressed integration sites characterized by very low burst sizes, but that this parameter regime might be more relevant for understanding reactivation from latency.

### Extension to a three-state model of the HIV LTR promoter with positive feedback

The additional reactions incorporated in a three-state model (Fig. 4B) are as follows:


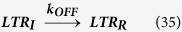



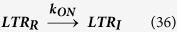


In **equations** 35 and 36, LTR_I_ refers to an ‘intermediate’ HIV promoter in the “OFF” state, and LTR_R_ refers to a ‘repressed’ promoter also in the “OFF” state. *k*_*ON*_ and *k*_*OFF*_ are parameters that account for the transition between the repressed and intermediate states. The reactions describing active to intermediate state transitions (i.e., **equations** 20 and 21) remain the same. Additionally, the reactions describing transcript and protein production/degradation (**equations** 1 through 4, 2, 3, 4) remain the same.

Recording reaction propensity and stoichiometry for the three-state model:


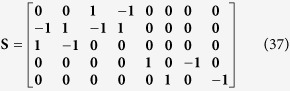



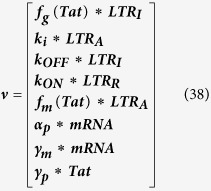


For the above model, the chosen parameters are listed in Table 1.

### Theoretical simulations

All simulations were performed in MATLAB (MathWorks, Inc.) and simulations were run on High Performance Computing (HPC) clusters at Yale University. For stochastic modeling, we implemented the Gillespie algorithm^51^ (see also tutorial^52^ as well as other computational studies implementing this approach^53^,^54^,^55^). All stochastic simulations were run for a period of 10 days. Mean protein counts, Fano factor, CV, and CV^2^ were computed from endpoint protein distributions; mean first passage time, mean onset time, and cell activation were computed from analyzing individual time traces. In all figures in the main article, the endpoint protein distributions were computed following 1000 independent Gillespie simulations (except when mentioned; specifically in Supplementary Figures S4, and S7–S13, metrics were computed after performing 100 independent Gillespie simulations as a representative sample).

## Experimental methods

Jurkat T cell clones J-Lat 8.4 and J-Lat 10.6 obtained from NIH AIDS Research and Reference Reagent Program, Division of AIDS, NIAID, NIH^56^ were used in Fig. 6C,D as examples of HIV integrated into repressed and permissive chromatin environments, respectively. Cells were cultured in Roswell Park Memorial Institute (RPMI) media 1640 supplemented with 10% fetal bovine serum, penicillin, streptomycin, and L-glutamine and grown at 37 °C and 5% CO_2_. Cells were maintained at 2 × 10^5^ cells/mL. Cells were grown to 5 × 10^5^ cells/mL and stimulated with indicated combinations of 5-aza-2′-deoxycytidine (Aza) (Sigma-Aldrich) and tumor necrosis factor alpha (TNF) (Peprotech). After 48 hours, cells were fixed in 4% formaldehyde. At least 10,000 cells were analyzed for LTR-driven GFP expression on an Accuri^TM^ C6 flow cytometer (BD Biosciences) for each condition.

## Additional Information

**How to cite this article**: Chavali, A. K. *et al.* Distinct promoter activation mechanisms modulate noise-driven HIV gene expression. *Sci. Rep.* **5**, 17661; doi: 10.1038/srep17661 (2015).

## Supplementary Material

Supplementary Information

## Figures and Tables

**Table 1:** Model parameters.

Parameters	Value	Reference
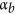	Varied from 0.01 to 10 day^−1^ (one-state); Varied from 0 to 48 day^−1^ (two- and three-state)	This study
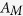	32	Within the range of values chosen in 19
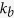	Varied from 0 to 7.2 day^−1^ (two- and three-state)	This study
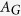	10	Within the range of values chosen in 19
K	100	Assumed
c	Varied from 0.01 to 100 (one-state); 1 (two- and three-state)	This study
q	Varied from 1 to 5 (one-state); 1 (two- and three-state)	41,53
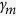	4.8 day^−1^	19
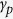	1.2 day^−1^	19
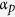	24 day^−1^	Approximated from 33
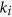	96 day^−1^ (two- and three-state)	19
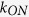	Varied from 0.01 to 10 day^−1^ (three-state)	This study
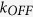	Varied from 0.01 to 10 day^−1^ (three-state)	This study
