# Supplementary material for: Distinct promoter activation mechanisms modulate noise-driven HIV gene expression
Source: Sci Rep. 2015 Dec 15;5:17661. doi: 10.1038/srep17661 (PMC4678399; doi:10.1038/srep17661)
Supplement: Supplementary Information [file srep17661-s1.pdf]

Distinct promoter activation mechanisms modulate noise-driven HIV gene expression

Arvind K. Chavali[1], Victor C. Wong[2], and Kathryn Miller-Jensen[1,2]*

[1]Department of Biomedical Engineering, Yale University
[2]Department of Molecular, Cellular, and Developmental Biology, Yale University
55 Prospect Street, New Haven, CT 06511

*Corresponding author: kathryn.miller-jensen@yale.edu

Supporting Figures and Tables

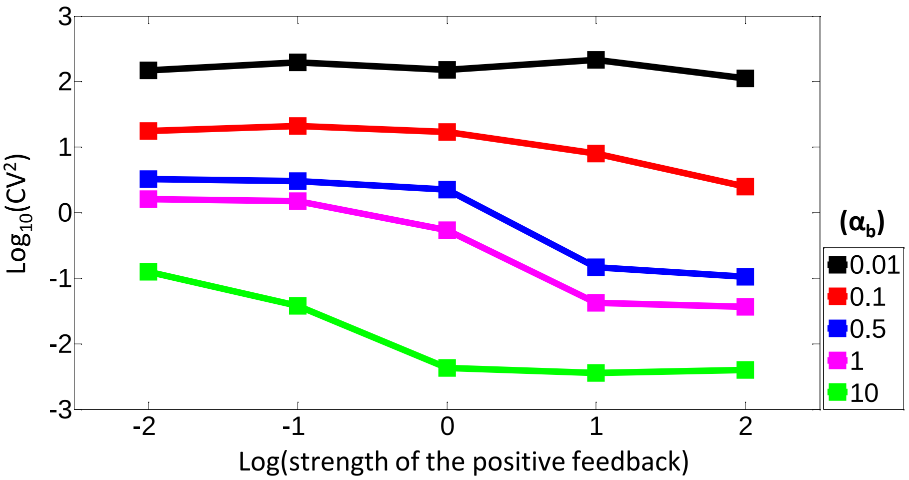

Supplementary Figure S1: Noise as measured by CV^2 in the one-state promoter model across varying basal transcription rates and strengths of positive feedback.

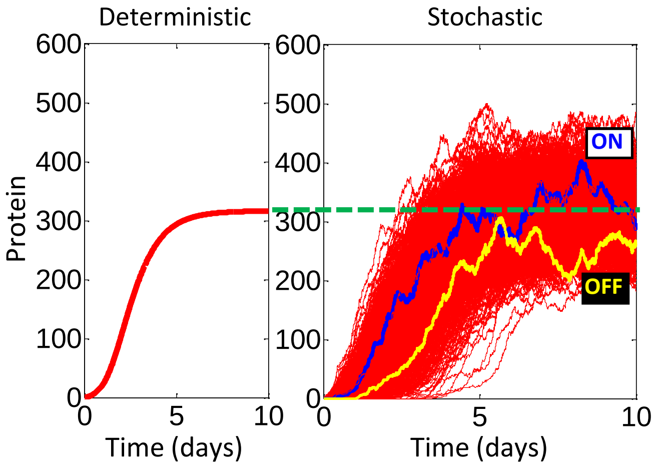

Supplementary Figure S2: The one-state promoter model provides a benchmark threshold for viral activation. The threshold for activation (green dotted line) was set at the deterministic steady-state protein value for an active one-state promoter system with positive (non-cooperative) feedback (left panel; deterministic). 1000 simulations of protein production in the stochastic model are presented in red (right panel; stochastic). Representative traces or cells that crossed the threshold (blue; “ON”) or that failed to cross the threshold (yellow; “OFF”) over time are shown.

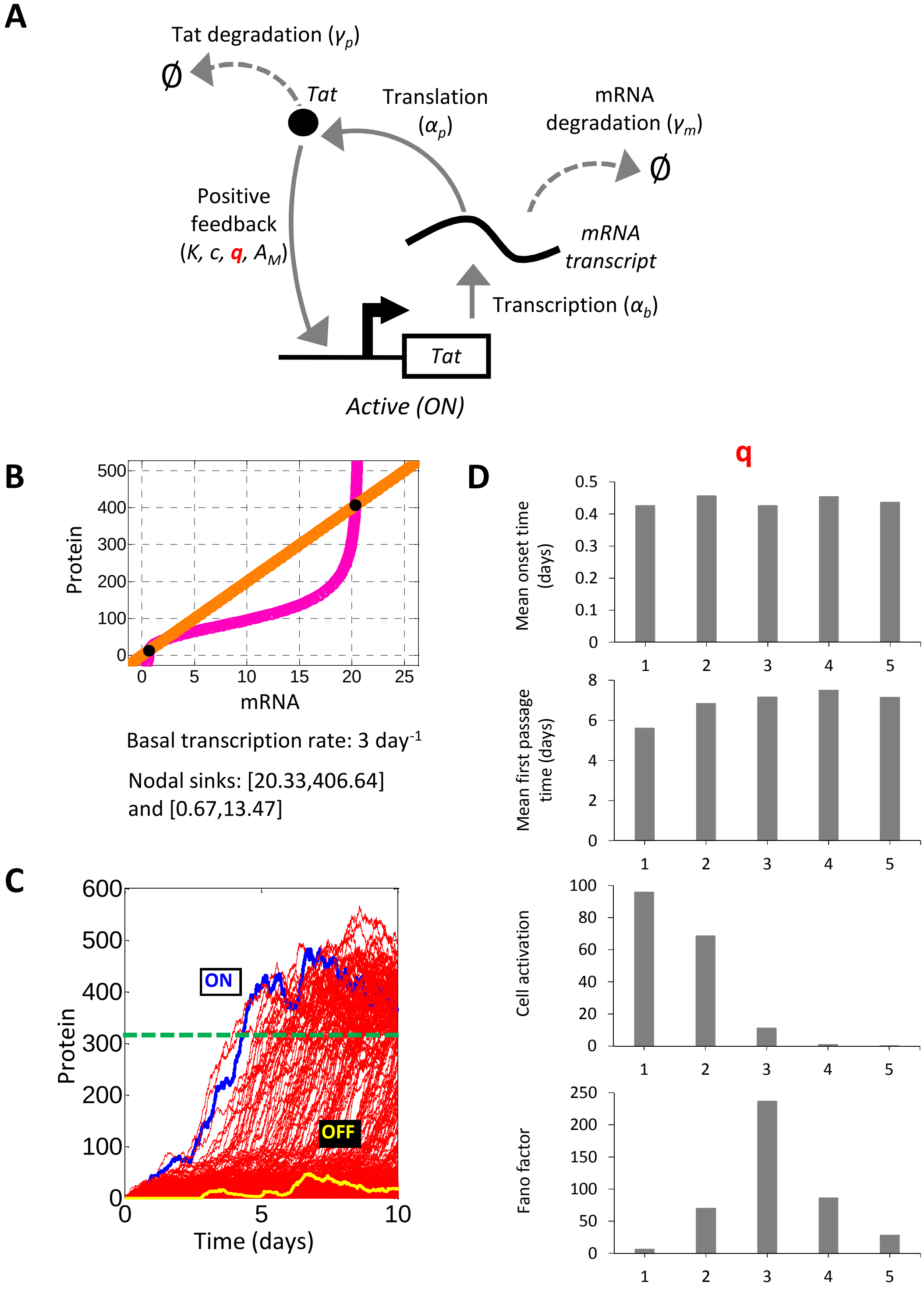

Supplementary Figure S3: Extended sensitivity analysis for a one-state model with positive feedback and exploration of cooperative behavior. **(A)** Schematic of model **(B)** Multiple nodal sinks and bistability in a one-state promoter model with cooperative feedback (q = 3) **(C)** 1000 simulations of protein production for a stochastic model of a one-state promoter system with cooperative feedback (q = 3). Representative traces or cells that crossed the threshold (blue; "ON") or that failed to cross the threshold (yellow; "OFF") over time are shown. The threshold for activation is presented as a green dotted line **(D)** The Hill co-efficient 'q' was varied from 1 to 5. For each simulation, mean onset time, mean first passage time, cell activation, and Fano factor are shown (for a representative set of n = 1000 runs).

A

B

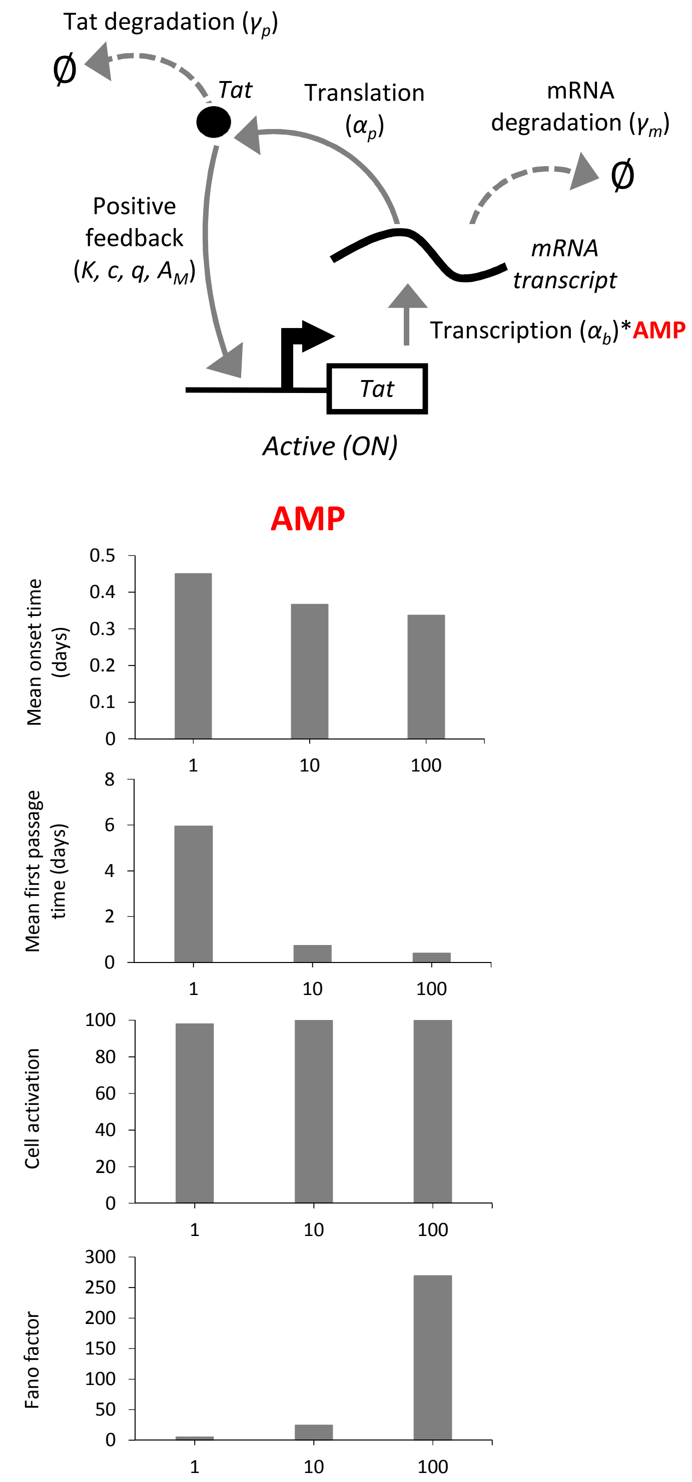

Supplementary Figure S4: Extended sensitivity analysis for a one-state model with transcriptional amplification. (A) An amplification factor (AMP) was introduced for every transcription event (see Methods) (B) For each simulation, mean onset time, mean first passage time, cell activation, and Fano factor are shown (for a representative set of n = 100 runs).

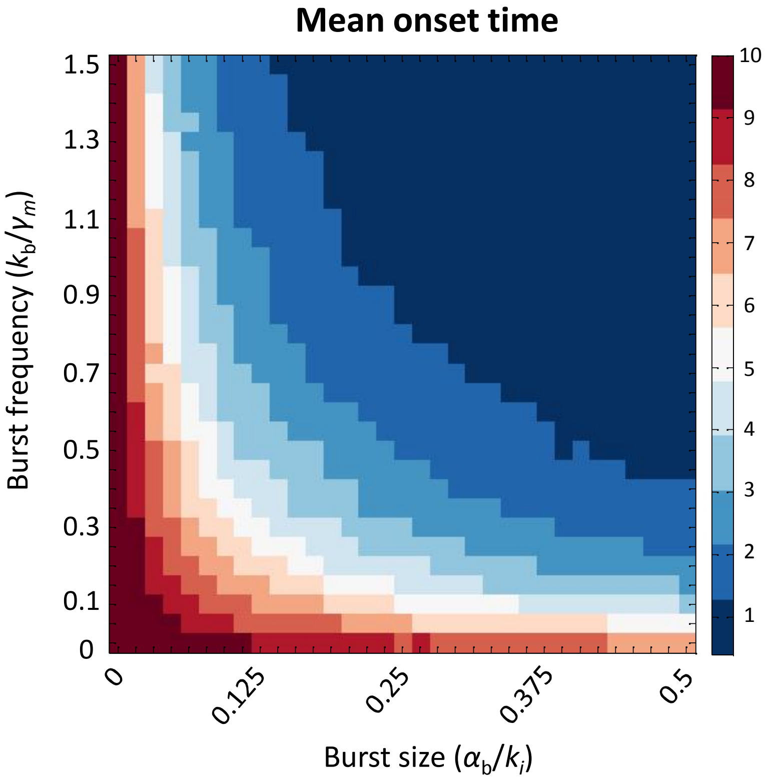

Supplementary Figure S5: Mean onset time (days) for a two-state promoter model under varying transcriptional burst sizes and frequencies.

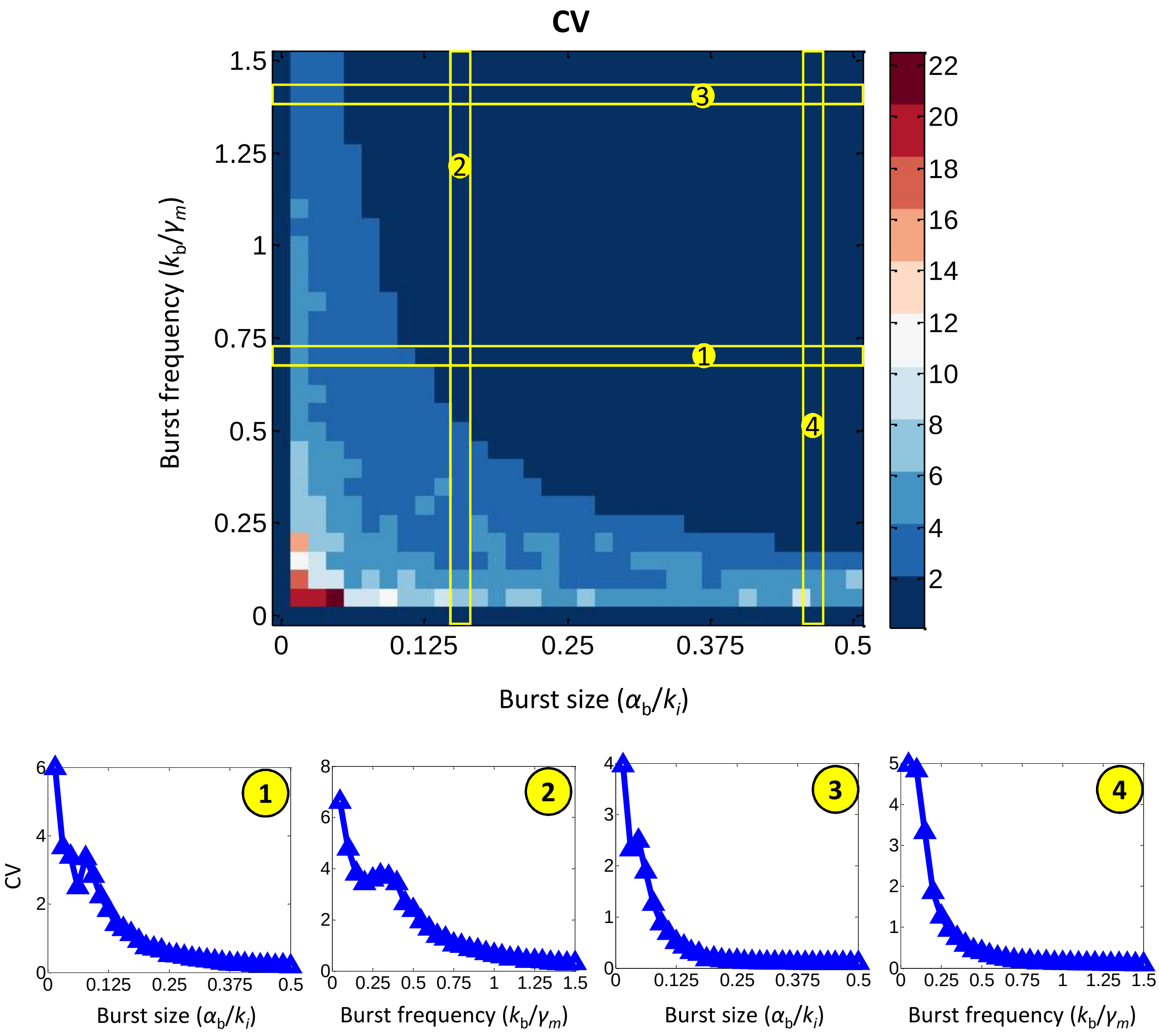

Supplementary Figure S6: Charting paths through CV for a two-state promoter model with positive feedback. These paths (i.e., lines of constant burst size or burst frequency) correspond to the same paths explored in Figure 3 (main text).

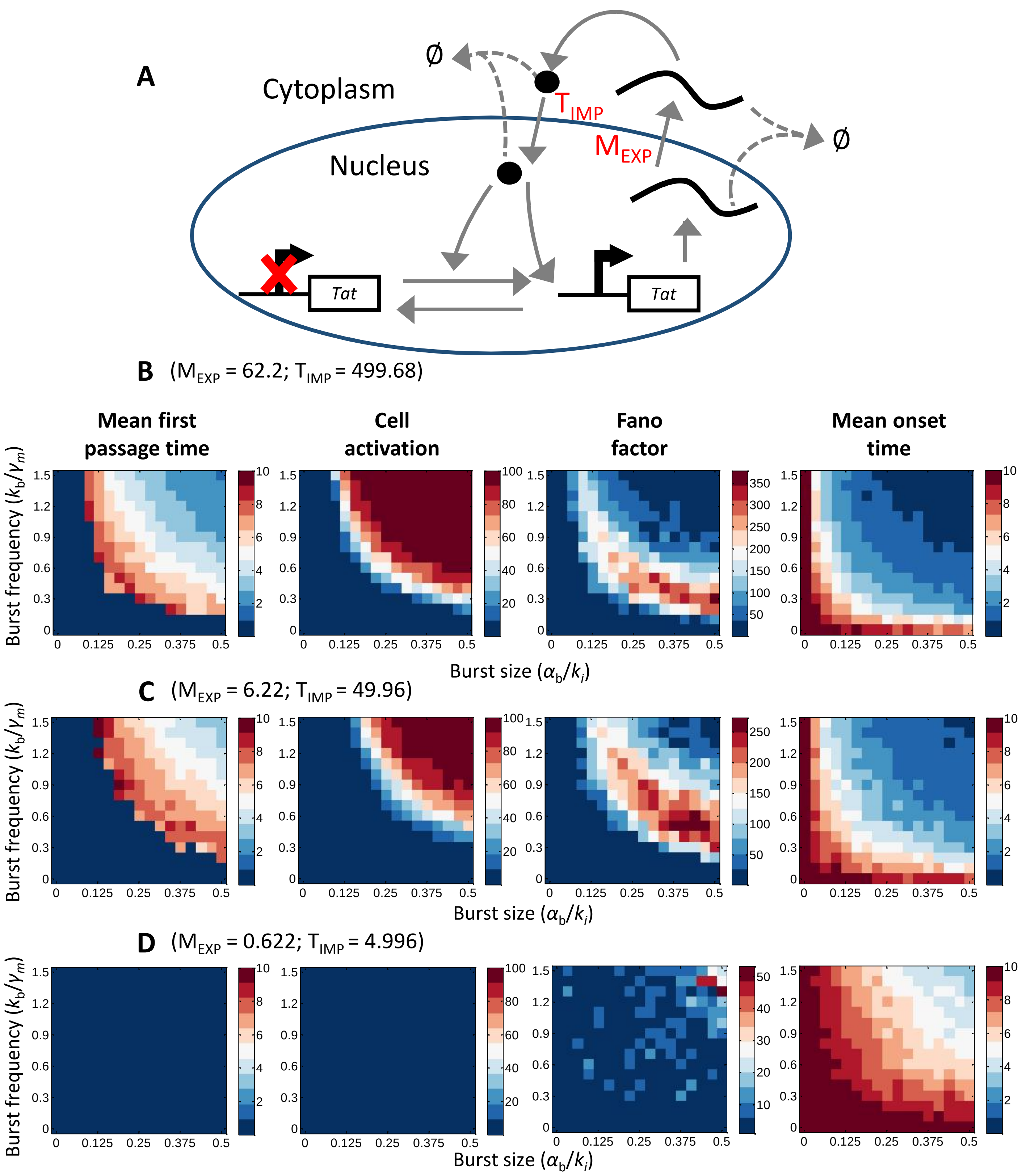

Supplementary Figure S7: Accounting for mRNA export from the nucleus and Tat import into the nucleus. **(A)** Schematic of model **(B)** Mean first passage time, cell activation, Fano factor, and mean onset time are shown (for a representative set of n = 100 runs) at a mRNA export rate (M_{EXP}) of 62.2 day^{-1} and a Tat import rate (T_{IMP}) of 499.68 day^{-1}. Same metrics are additionally shown for **(C)** M_{EXP} of 6.22 day^{-1} and T_{IMP} of 49.968 day^{-1}, and **(D)** M_{EXP} of 0.622 day^{-1} and T_{IMP} of 4.996 day^{-1}. Altering these rates can lead to significant variations in onset time of Tat production. X and Y axes of the heat plots indicate variations in transcriptional burst size and burst frequency, respectively.

Mean protein counts for a 'two-step' three-state promoter model

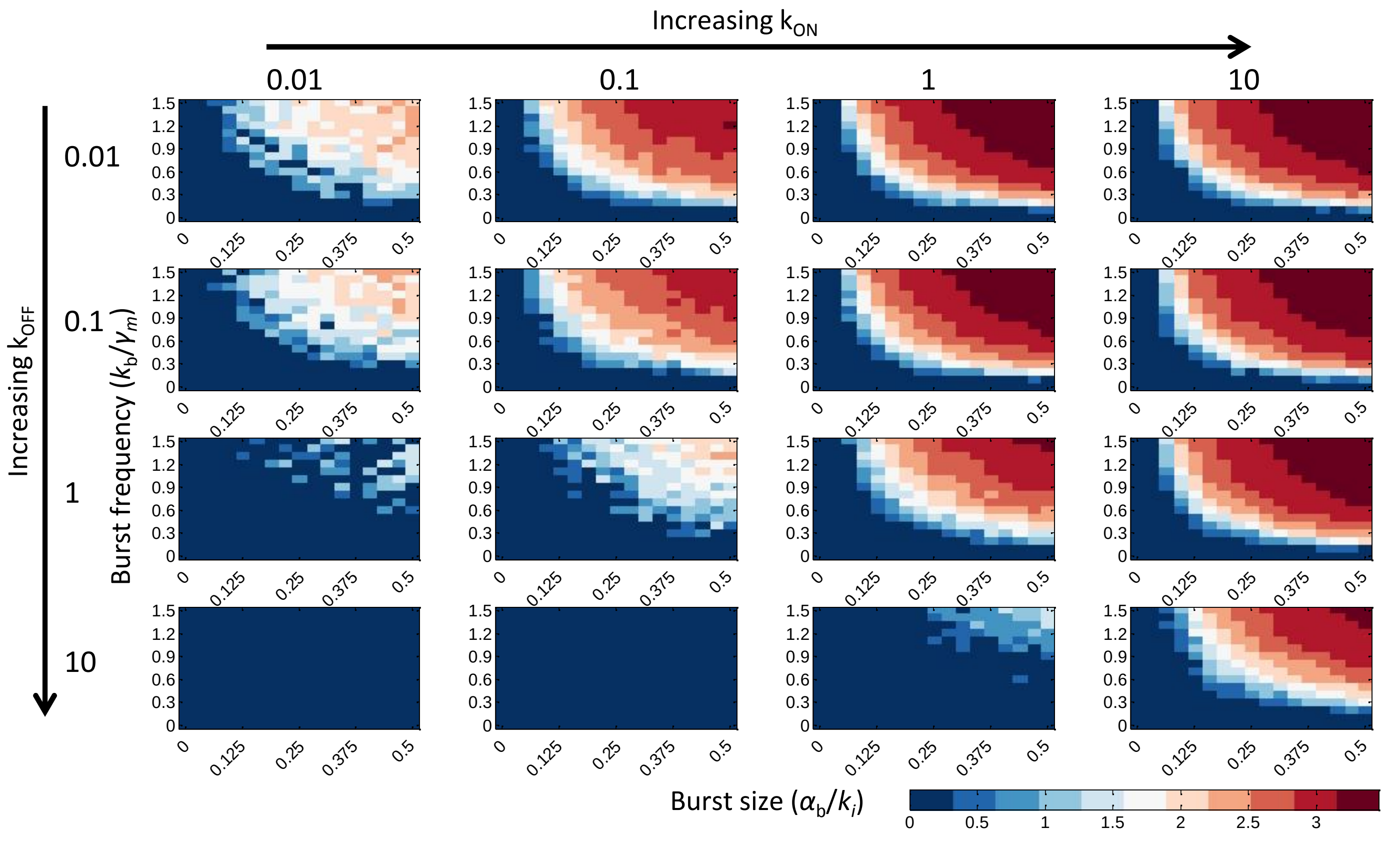

Supplementary Figure S8: Mean protein counts is plotted for a 'two-step' three-state model (with an intermediate state transition). k_{ON} and k_{OFF} rates were varied from 0.01 to 10 day^{-1}, and the model was simulated under different conditions of burst size and frequency (for a representative set of n = 100 runs). Color scale is plotted in log units.

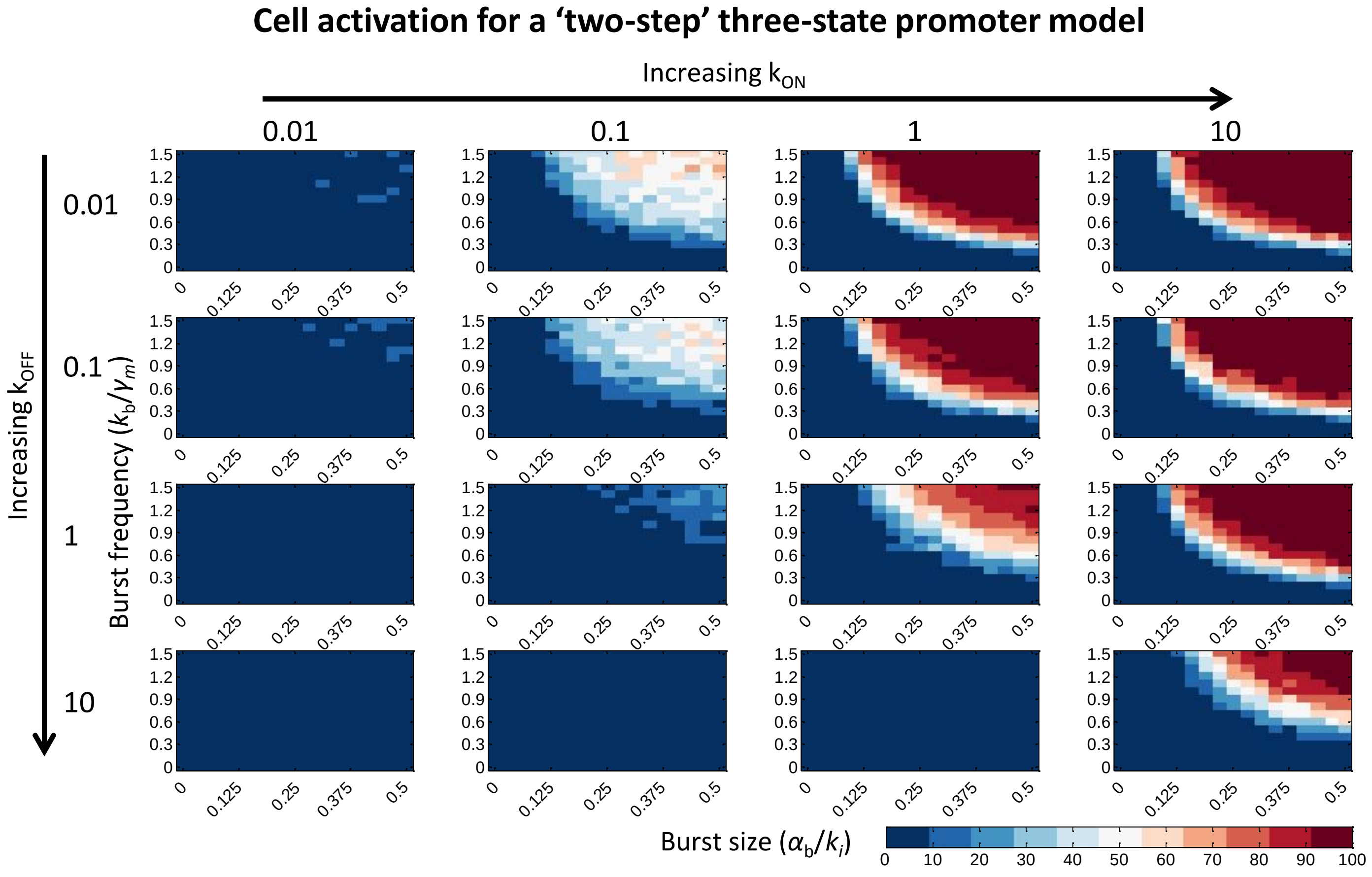

Supplementary Figure S9: Cell activation is plotted for a 'two-step' three-state model (with an intermediate state transition). k_{ON} and k_{OFF} rates were varied from 0.01 to 10 day^{-1}, and the model was simulated under different conditions of burst size and frequency (for a representative set of n = 100 runs).

Fano factor for a 'two-step' three-state promoter model

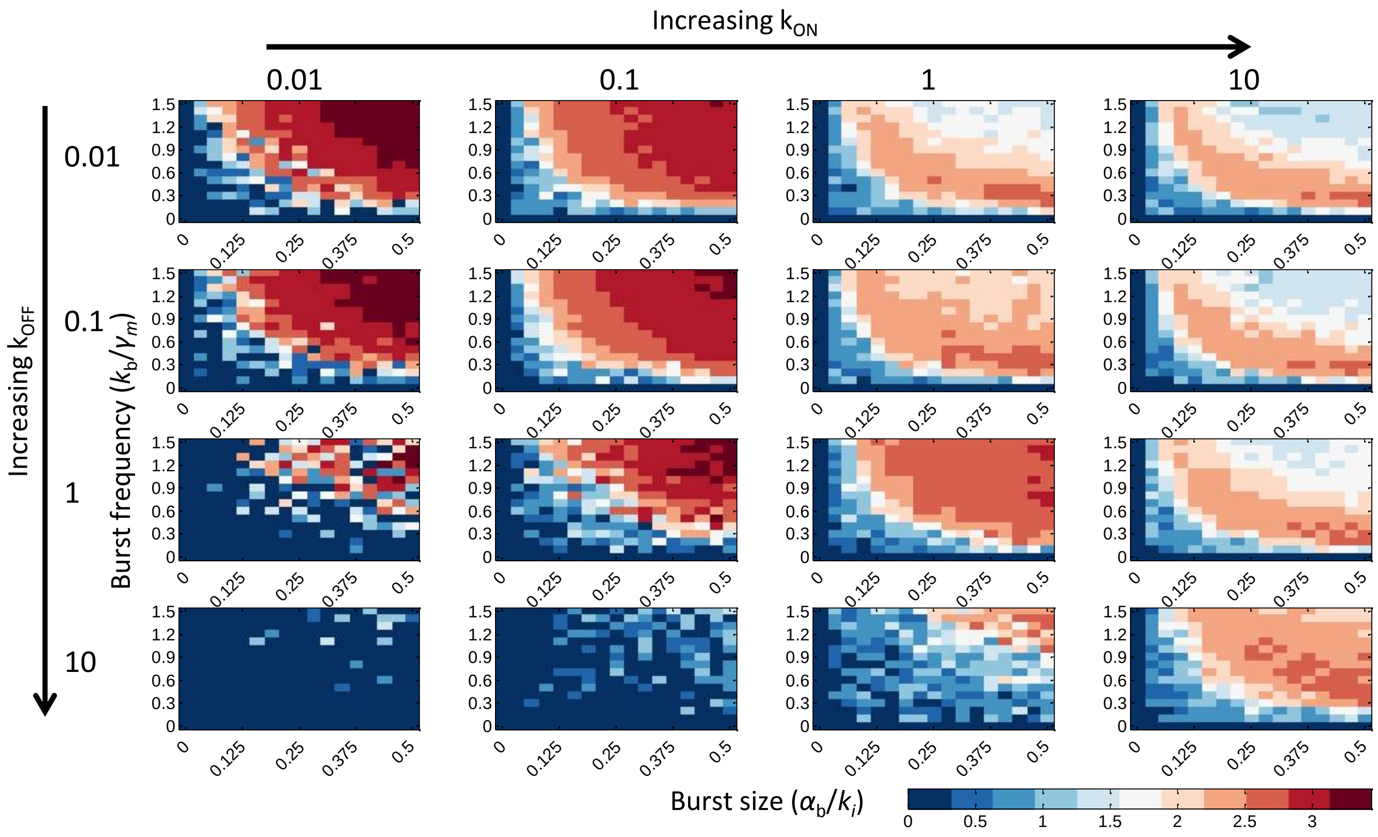

Supplementary Figure S10: Fano factor is plotted for a 'two-step' three-state model (with an intermediate state transition). k_{ON} and k_{OFF} rates were varied from 0.01 to 10 day^{-1}, and the model was simulated under different conditions of burst size and frequency (for a representative set of n = 100 runs). Color scale is plotted in log units.

Mean first passage time for a ‘two-step’ three-state promoter model

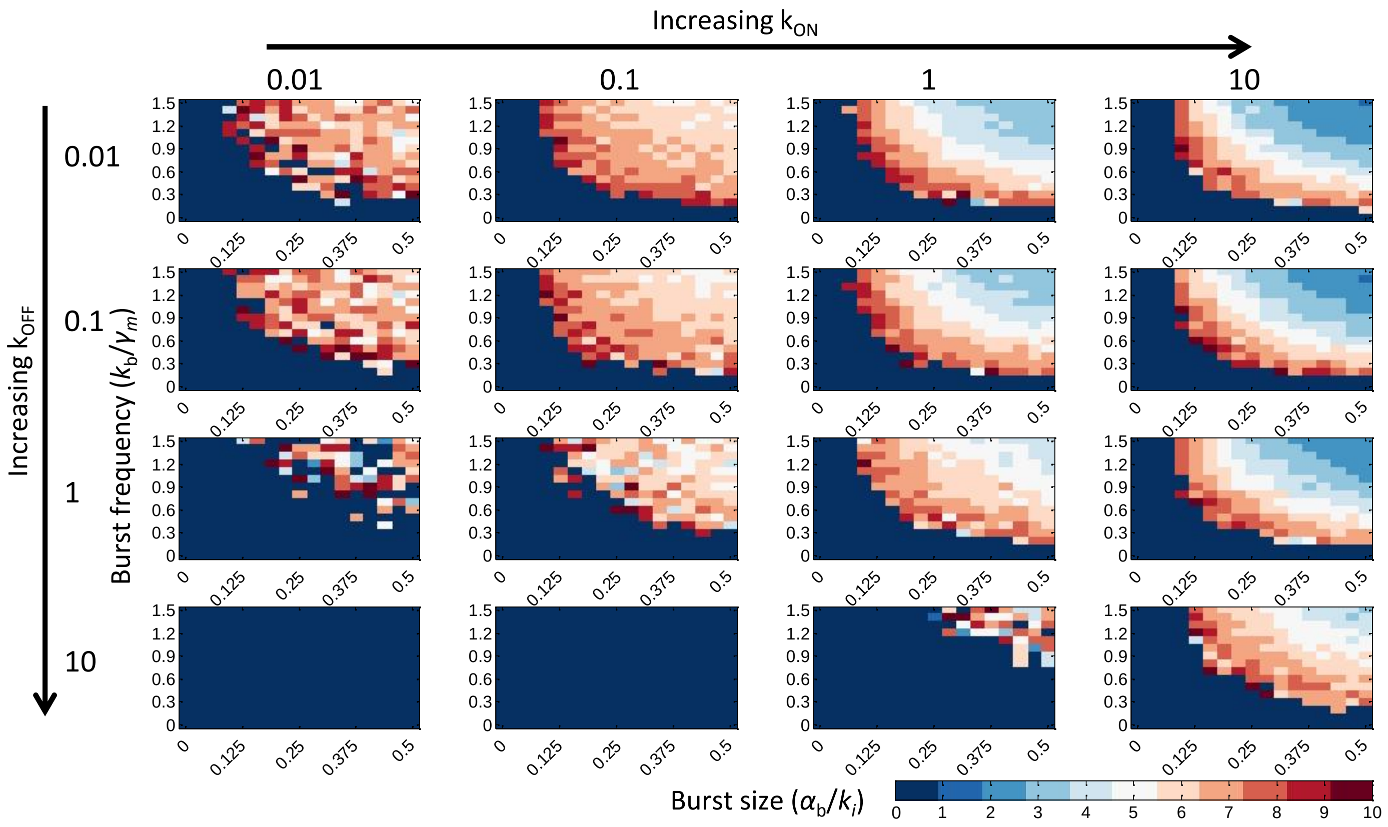

Supplementary Figure S11: Mean first passage time (days) is plotted for a ‘two-step’ three-state model (with an intermediate state transition). k_{ON} and k_{OFF} rates were varied from 0.01 to 10 day^{-1}, and the model was simulated under different conditions of burst size and frequency (for a representative set of n = 100 runs).

Influence of Tat positive feedback in a two-state promoter model

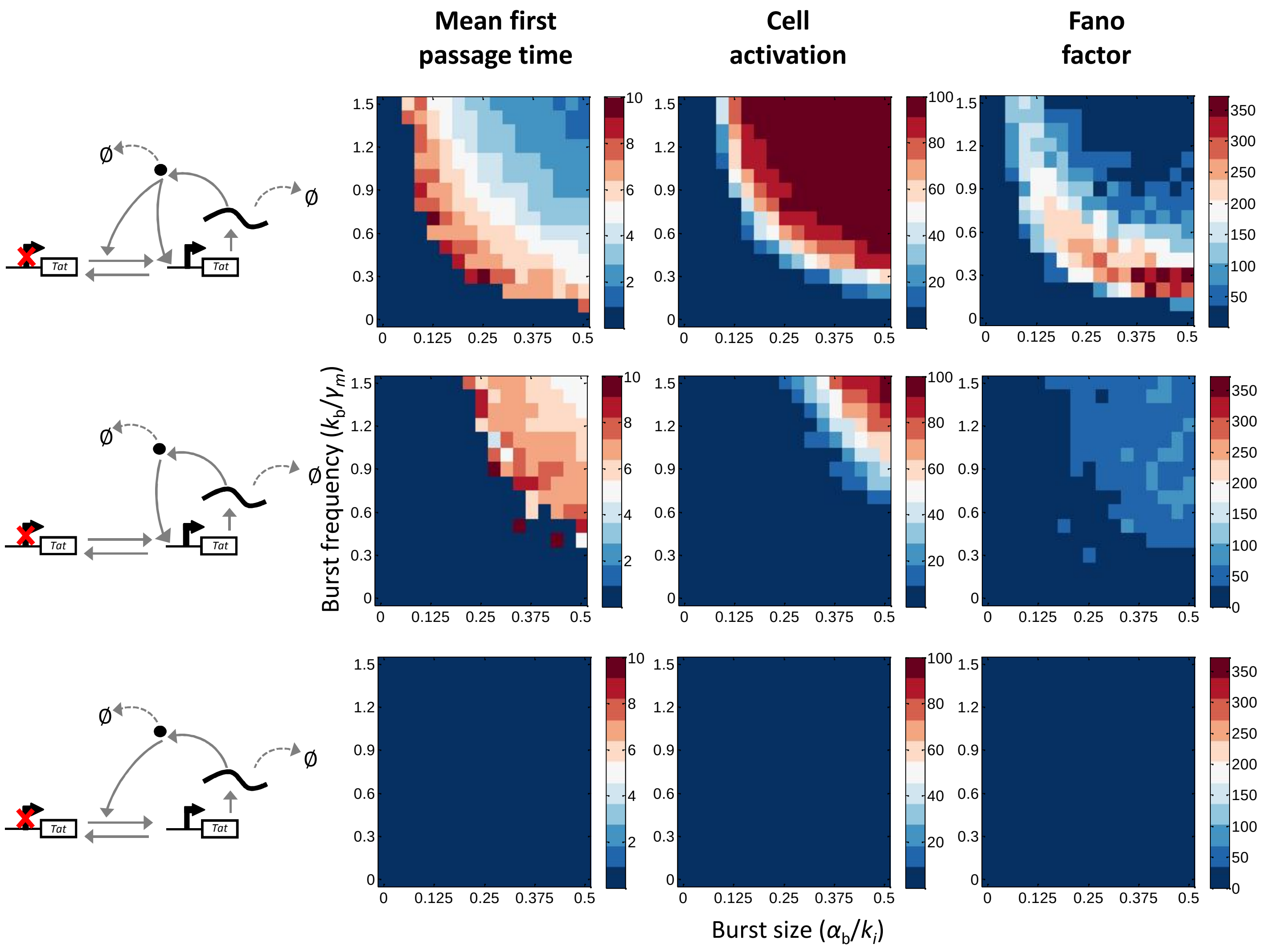

Supplementary Figure S12: Variations in Tat positive feedback behavior for a two-state promoter model. Mean first passage time, cell activation, and Fano factor are shown (for a representative set of n = 100 runs). X and Y axes of the heat plots indicate burst size and burst frequency, respectively.

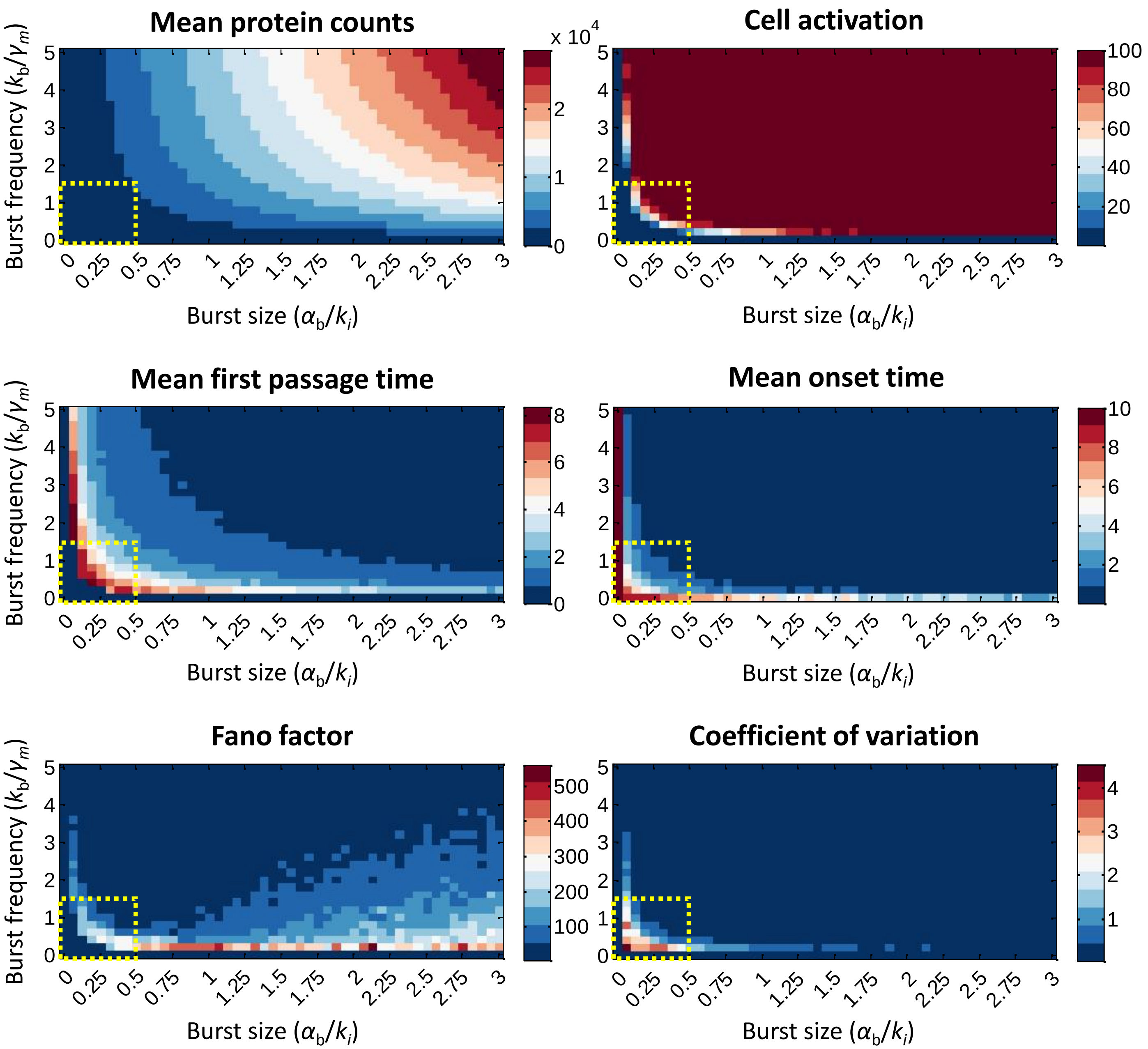

Supplementary Figure S13: Extending the range of burst size and frequency for a two-state promoter model with positive feedback (for a representative set of n = 100 runs). The yellow dotted box indicates the sampled ranges in the main text.

Reference	Burst size	Burst frequency	Mean no. of transcripts
Skupsky et al., PLoS Comp Bio, 2010	1.8 – 40	0.3 – 3.2	
Dar et al., PNAS, 2012	100 – 300	0.3 – 1	~110
Dey et al., Mol Syst Biol, 2015	2 – 23	0.7 – 4.2	4 – 38

Supplementary Table S1: Experimental values of burst size and frequency.
